# CASSPER is a semantic segmentation-based particle picking algorithm for single-particle cryo-electron microscopy

**DOI:** 10.1038/s42003-021-01721-1

**Published:** 2021-02-15

**Authors:** Blesson George, Anshul Assaiya, Robin J. Roy, Ajit Kembhavi, Radha Chauhan, Geetha Paul, Janesh Kumar, Ninan S. Philip

**Affiliations:** 1 Artificial Intelligence Research and Intelligent Systems (airis4D), Thelliyoor, Kerala India; 2 Department of Physics, CMS College, Kottayam, Kerala India; 3 Laboratory of Membrane Protein Biology, National Centre for Cell Science, S. P. Pune University Campus, Pune, India; 4 Inter-University Centre for Astronomy and Astrophysics (IUCAA), S. P. Pune University Campus, Pune, India; 5 Laboratory of Structural Biology, National Centre for Cell Science, S. P. Pune University Campus, Pune, India

**Keywords:** Cryoelectron microscopy, Data processing

## Abstract

Particle identification and selection, which is a prerequisite for high-resolution structure determination of biological macromolecules via single-particle cryo-electron microscopy poses a major bottleneck for automating the steps of structure determination. Here, we present a generalized deep learning tool, CASSPER, for the automated detection and isolation of protein particles in transmission microscope images. This deep learning tool uses Semantic Segmentation and a collection of visually prepared training samples to capture the differences in the transmission intensities of protein, ice, carbon, and other impurities found in the micrograph. CASSPER is a semantic segmentation based method that does pixel-level classification and completely eliminates the need for manual particle picking. Integration of Contrast Limited Adaptive Histogram Equalization (CLAHE) in CASSPER enables high-fidelity particle detection in micrographs with variable ice thickness and contrast. A generalized CASSPER model works with high efficiency on unseen datasets and can potentially pick particles on-the-fly, enabling data processing automation.

## Abstract (web summary)

George, Assaiya et al. develop a deep learning tool, CASSPER, that automates the detection of protein particles in transmission microscope images. This algorithm uses semantic segmentation and visually prepared training samples to capture the differences in the transmission intensities of microscope images, enabling automation of data processing.

## Introduction

Single-particle cryo-electron microscopy (cryo-EM) has revolutionized the field of structural biology by facilitating the structure determination of various biological macromolecules and their complexes^1–4^, which were recalcitrant to structure determination by X-ray crystallography or were not suitable for structure determination via NMR. Cryo-EM enables structure determination of proteins in solution without the need for protein crystallization or limitations of size, making it the current method of choice. A number of research projects are currently being carried out around the world to further improve the hardware^5–7^ and software^8–12^ in order to streamline and automate the data collection and processing steps for 3D structure determination. One of the obstacles that still remains for automating the single-particle cryo-EM structure determination is the manual identification and selection of particles (protein) from micrographs for extraction and subsequent 2D classification.

To achieve a high-resolution protein structure, the selection of a large number of good-quality particles is the prime requisite. However, particle identification, picking, and selection is a tedious and challenging process due to the increasingly larger datasets that are being collected nowadays. Moreover, the low signal-to-noise ratio (SNR) of the micrographs, presence of contaminants, contrast differences owing to varying ice thickness, absence of well-segregated particles, etc. further increase the difficulty levels. To overcome the above-mentioned drawbacks introduced primarily by EM grid vitrification and low dose imaging, one often has to rely on manual or semi-automated methods of particle-picking which could be slow and laborious for large-sized datasets. A fast, automatic method that can replace the manual processing is thus a necessity for automating the structure determination process.

Presently, considerable effort is being devoted to the development of automated particle picking methods in order to circumvent the manual intervention. These can be broadly categorized into two groups: (i) template-free and (ii) template-based methods, which rely mainly on cross-correlation with the template images. Gautomatch^13^ is one of the widely used methods for particle picking from cryo-EM micrographs with or without templates. In RELION^11^ and cryoSPARC^12^, a Gaussian blob of defined size is used as a template for particle picking. Similarly, DoGpicker^14^ uses mathematically derived Gaussian functions as templates to recognize and select particles from the micrographs. However, these tools are prone to pick huge amounts of contaminants, background, and ice, and do not work optimally for datasets with poor SNR or small particle sizes. These problems are resolved to a certain extent in template (reference)-based particle picking tools implemented in SIGNATURE^15^, RELION^11^, cryoSPARC^12^, EMAN^8^, SPHIRE^16^, cisTEM^17^, FindEM^18^, gEMpicker^19^, SPIDER^20^, etc. In all these methods, templates are generated by manually picking a few hundred to several thousand particles from multiple micrographs. These particles are then sorted, and 2D classified to generate templates for automated particle selection via template matching algorithms. While this methodology works better than previously described reference-free methods, it is time-consuming, computationally expensive, and also requires manual intervention preventing its integration into automated pipelines for structure determination. Besides, manual particle picking may introduce a strong template bias, especially for asymmetric molecules that may result in a high false picking rate.

Artificial intelligence/machine learning (AI/ML)-based approaches have the potential to overcome the problems discussed above and pave the way for full-automation of the data processing pipeline. Not surprisingly, multiple AI/ML-based methods have already been proposed, such as XMIPP^21^, APPLE picker^22^, DeepPicker^23^, DeepEM^24^, FastParticle Picker^25^, crYOLO^26^, PIXER^27^, PARSED^28^, WARP^29^, Topaz^30^, AutoCryoPicker^31^, etc. that are based on Convolutional Neural Networks (CNN), region-based Convolutional Neural Networks (R-CNN), cross-correlation, and segmentation. These deep learning classifiers are first trained on available datasets with known labels. The training process allows the classifiers to learn intrinsic and unique features/characteristics of the particles. The trained classifier can then be used to pick similar particles from other micrographs automatically. Boxnet implemented in WARP uses ResNet architecture and predicts particles based on the argmax operations. In CNN-based methods like DeepPicker, DeepEM, and Topaz, a sliding window is used to analyze the image for classification. In crYOLO, which is also a CNN-based tool, the entire image is split into grids, and part of the image in each grid is taken as an input to the classifier. However, all the above methods require individual particles to be manually picked for training, which is a time-consuming procedure. Further, the exposure difference, noise level, and the variable ice thickness in micrographs also limit the performance of automated particle picking tools.

Here, we present a method packaged as CASSPER (Cryo-EM Automatic Semantic Segmentation-based Particle pickER) based on semantic segmentation (SS) for automated particle picking with high precision and accuracy. To our knowledge, CASSPER is the first deep learning-based tool that does not require manual particle picking to train and predict different kinds of particles (protein, ice, carbon, etc.) in an EM micrograph. Employing SS, CASSPER learns how to differentiate each pixel of the image by considering the difference in the scattering intensity of the particles in the medium. Since protein, ice, and carbon contamination differ in scattering intensity, CASSPER can differentiate between them and provide unique labels with high confidence and reliability. CASSPER learns at the pixel level rather than just relying on the shapes of the particle to yield high accuracy on even unseen micrographs. CASSPER has a graphical user interface (GUI) with a few track (slide) bars that can be adjusted to label all the particles in a micrograph in one go, making it highly efficient and time-saving in the preparation of the training data. This unique feature that eliminates manual picking of particles distinguishes CASSPER from all other existing methods. For removing regional contrast variability in micrographs, CASSPER utilizes the Contrast Limited Adaptive Histogram Equalization (CLAHE)^32^ algorithm. CLAHE implements adaptive histogram equalization by dividing the image into grids of small areas called “tiles”. To smoothen the boundaries, adjacent tiles are combined using bilinear interpolation. The contrast limiting feature, which is unique in CLAHE, eliminates noise amplification in an image by redistributing the excess pixel values in regions with high contrast to the neighboring pixels.

## Results

### Implementation of SS in CASSPER

Unlike the traditional image classification methods that use either derived features or morphological characteristics of the target image for its identification, CASSPER uses SS for identifying the protein molecules at the pixel level^33^. CASSPER is coded in Python and employs InceptionV4^34^ for feature extraction and FRRN (Full Resolution Residual Network)^35^ architecture for SS.

**Fig. 1:**
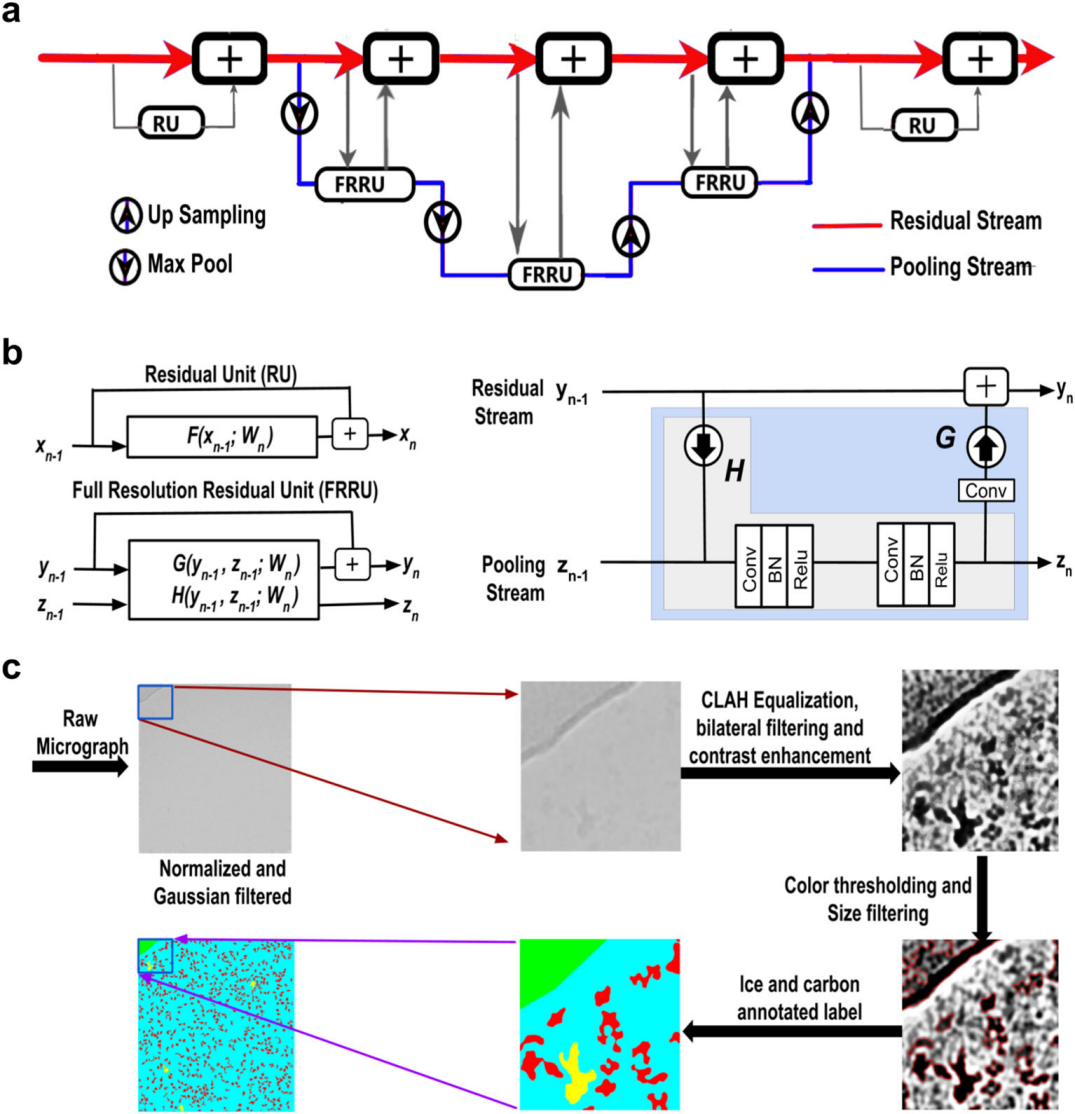
Structure of full resolution residual network (FRRN) and the flowchart depicting the labeling tool. **a** Abstract structure of full resolution residual network (FRRN). FRRN achieves better recognition and localization performance by image processing in two different streams; namely, pooling and residual streams. Pooling stream learns the abstract relationships in the image and the residual stream carries a full-resolution feature map that ensures localization capability. **b** The detailed structure of FRRU. The schematic representation of FRRU and RU is also shown. FRRU combines the pooling and residual stream and returns it to the next layer. A Convolutional layer, Batch normalization, and Relu activation layer is performing the pooling operation in FRRU. **c** Flowchart of the image-labeling tool which is used for preparing training data for Semantic Segmentation. The motion corrected mrc is filtered using a Gaussian filter after normalization. This step makes the micrograph more visible for the user. Contrast Limited Adaptive Histogram Equalization (CLAHE) is applied to eliminate any exposure differences in the image and bilateral filtering followed by contrast enhancement makes the difference between background and particles more vivid. Intensity thresholding is done to distinguish particles from background. Size thresholding is done for removal of contaminants or background that is not eliminated at intensity thresholding. The pixels corresponding to proteins are indicated in red. The ice and carbon contaminations are later labeled selecting the regions of interest.

The FRRN has two different processing streams, namely, a full-resolution residual stream that holds high-resolution details for recovering the location of the detections and a pooling stream for extracting the hidden features required for learning the abstract relationships in the image. By using a set of Full Resolution Residual Units (FRRU) to merge the residual stream and the information from the pooling layers at each stage, localization, as well as classification accuracy during reconstruction, is ensured. This is one advantage of using FRRN instead of the more popular Fully Convolutional Neural Network (FCN). The network architecture of FRRN is shown in Fig. 1a. The Residual Network^36^ in FRRN is composed of a series of Residual Units (RUs). Each RU has one input and one output. The skip connection structure in RU reduces the vanishing gradient problem and increases the training accuracy. The output of the *n*th layer of the RU is given by

$$x_n = x_{n - 1} + F\left( {x_{n - 1};W_n} \right)$$

where *F*(*x*_*n*−1_; *W*_*n*_) is the Residual of the layer.

The FRRU has two inputs and two outputs. The outputs of the *n*th layer *y*_*n*_ and *z*_*n*_ of the FRRU has two functions *G* and *H* such that

$$y_n = y_{n - 1} + G\left( {y_{n - 1},\,z_{n - 1};\,W_n} \right)\,\left( {{\mathrm{residual}}\,{\mathrm{stream}}} \right)$$

$$z_n = H\left( {y_{n - 1},\,z_{n - 1};\,W_n} \right)\,\left( {{\mathrm{pooling}}\,{\mathrm{stream}}} \right)$$

where *y*_*n*−1_ and *z*_*n*−1_ are the residual and pooling inputs to FRRU. If the pooling function *H* is zero, then the FRRU reduces to RU. The FRRN architecture that is used for the present study employs five FRRUs for upsampling and four FRRUs for downsampling. Also, it has five max-pooling and four unpooling layers in the pooling stream. The detailed structure of the FRRU is shown in Fig. 1b. CASSPER uses the SS implementation by George Seif^37^.

### CASSPER pipeline and preparation of training data

**Fig. 2:**
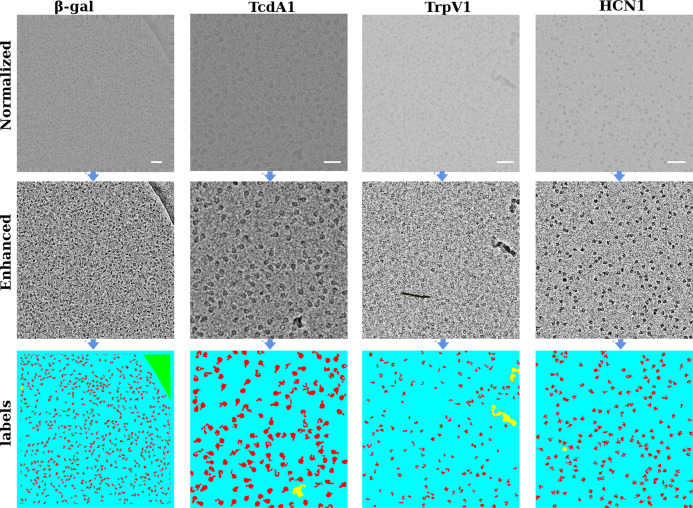
From raw to semantically segmented micrographs. The raw micrographs, contrast enhanced and semantically segmented images of micrographs from β-galactosidase (EMPIAR 10017), TcdA1 (EMPIAR 10189), TRPV1 (EMPIAR 10005), and HCN1 (EMPIAR 10081), respectively. The entire micrograph is segmented into different classes and each class is represented by a unique color. The background, protein, ice contamination, and carbon contamination are represented using cyan, red, yellow, and green colors, respectively. Scale bar is set at 50 nm.

**Fig. 3:**
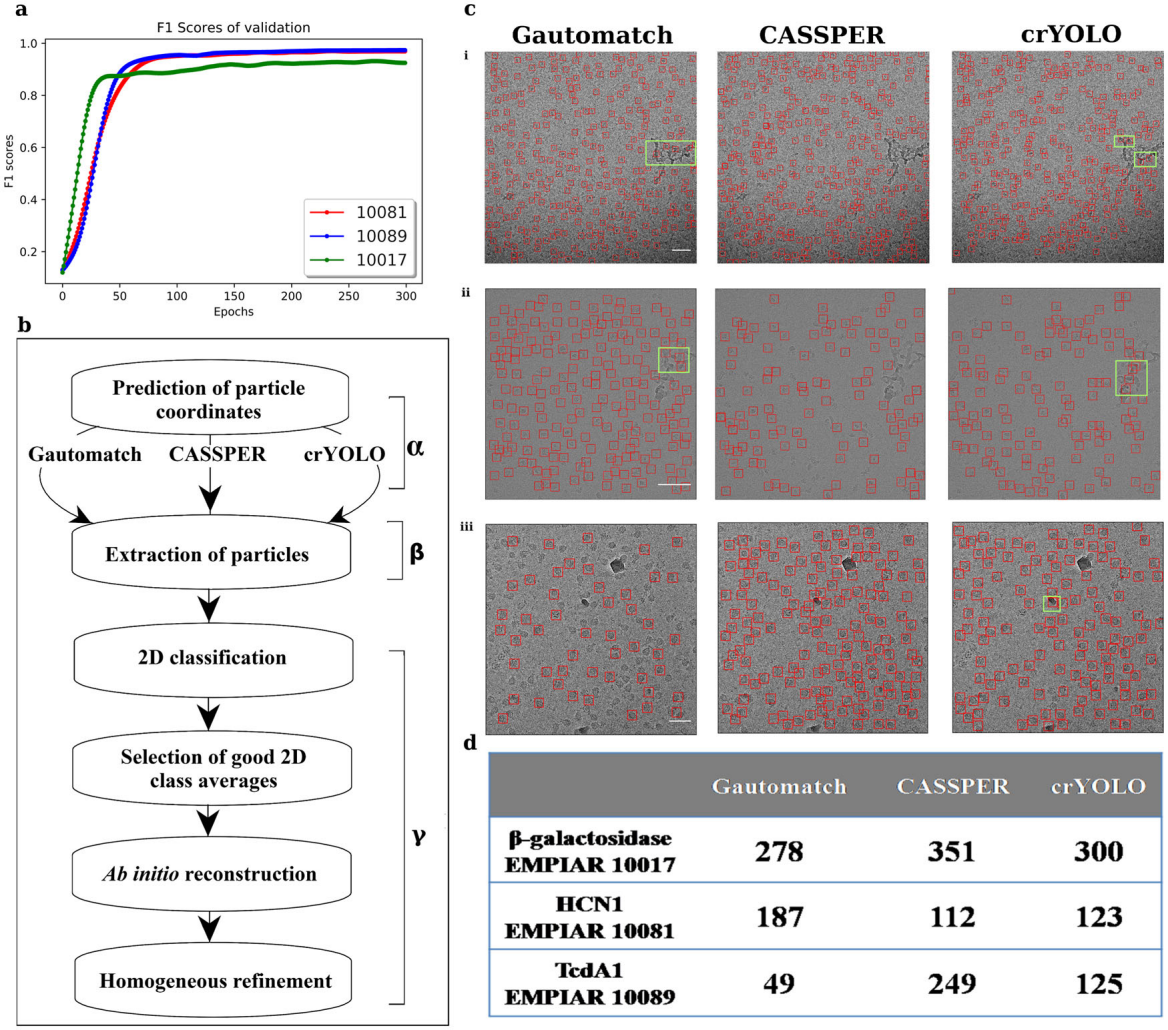
Training and picking by CASSPER and comparison with other tools. **a** The validation F1 scores, which are used to select the best trained model in training epochs for prediction, for all three proteins. **b** Schematic representation of the uniform pipeline used to compare the performance of Gautomatch, CASSPER, and crYOLO. **c** Representative micrographs (scale bar, 50 nm) for (i) β-galactosidase, (ii) HCN1, and (iii) TcdA1 showing the particle picking performance of different tools. Highlighted areas indicate the noise picked by the respective tools. **d** Table showing the number of particles picked by Gautomatch, CASSPER, and crYOLO on the same micrograph for each protein as shown in **c**.

The entire pipeline of CASSPER can be divided into two phases—the training phase and the prediction phase. Like all other supervised ML methods, SS also requires training data. The training data are prepared by labeling the different objects of interest in the image by assigning them unique colors. Thus, the label itself is an image with colored pixel mask indicating their type. Since it is a SS-based learning method, all the different object types must be labeled. In order to carry out labeling with minimum user intervention, we developed a graphical labeling tool. The tool enables visual enhancements in the image by varying its contrast, bilateral filter size, intensity, and threshold values. All controls are easily implemented by adjustments of the track bars as explained in the “Methods”. A schematic of the functionalities of the track bars are shown in Fig. 1c. The method is independent of the structural details of the protein, and hence its efficiency is unaltered by the differences in shape or size of the projected image of the protein. An illustration of that is shown in Fig. 2, where the micrographs are labeled to show four different constituents (referred to as classes hereafter), namely crystalline ice, carbon edges, background, and the protein molecules. About 12–20 micrographs of each protein were labeled and used to train the network. The raw and labeled micrographs for training were provided with the same root names in the pipeline, and about 80% of them were cycled in ~300 epochs to train the network. The remaining 20% of the data was used for validating the performance of the trained network. The training round that gives the best F1 scores (see section “Statistics and reproducibility” for details) during validation was taken as the criterion for choosing the final trained model. Subsequent prediction on the larger set of unlabeled micrographs was done using the trained model. F1 scores for three proteins during validation are shown in Fig. 3a as an example. CASSPER labels each particle with the same colors that were used to represent them in the training data. Since we are interested only in finding the coordinates of the protein, everything other than the protein is masked out from the image. The user is then allowed to specify the size of a circular mask approximately the size of the protein. This input is needed as occasionally the image of the protein may appear fragmented, and the machine needs this information to include those fragments as part of the same particle. CASSPER then estimates the centers of those contours, and its coordinates are provided in the popular box and star format for particle extraction and subsequent processing steps.

### Uniform pipeline for comparison

The performance of CASSPER was evaluated by comparing the results with two popular particle picking tools—ML-based crYOLO and cross-correlation-based Gautomatch. A proper comparison of the performance of CASSPER with crYOLO and Gautomatch requires a common platform, and hence we designed a uniform pipeline where the particles predicted by these tools are subjected to a minimum number of processing steps. The goal of using this pipeline is to allow the particles predicted by these tools to pass through precisely the same course of data-processing steps and assess the results. However, it is to be noted that the resolutions achieved through this uniform scheme could be further improved by including more number of iterative 2D classification and refinement steps. Since our goal was to compare picked particles from all the three tools, we have limited the number of steps of data processing. The uniform pipeline scheme can be divided into three phases, as illustrated in Fig. 3b. Phase **α** predicts the particle coordinates using Gautomatch, CASSPER, and crYOLO. The same set of micrographs were used for particle prediction by all the three tools on four datasets discussed later (“CASSPER performance” section). A comparison for the true positive (actual protein particles) and false positive (wrong prediction as protein particles) on one representative micrograph for each protein as picked by Gautomatch, CASSPER, and crYOLO is shown in Fig. 3c, and the total number of particles picked by each tool is summarized in Fig. 3d. In this phase, each tool outputs a star file with the particle coordinates they detect. In Phase **β**, the particle coordinates were imported into RELION^11^ and the particles were extracted from the micrographs. The extraction box size was kept uniform for each protein predicted with different tools. Phase **γ** of the pipeline is carried out in cryoSPARC v1 and includes one single step of 2D classification, selection of 2D classes followed by ab initio 3D reconstruction, and a single step of homogenous refinement to accelerate the data processing. Thus, the extracted particle stacks obtained by the three tools for each of the four datasets (explained in the next section) were imported into cryoSPARC v1, where 2D classification was performed. After a single round of 2D classification, class averages with discernible features were selected and used for ab initio reconstruction with C1 symmetry. Later the 3D models generated from them were refined with single-step homogenous refinement in their respective symmetry groups. The number of good 2D classes and particles therein and the resolution of the map in subsequent 3D reconstruction achieved, indirectly reflect the performance of the particle picking tools (Supplementary Table 1).

We trained crYOLO for each dataset, and the trained model was used to predict the particles for comparison. In the case of Gautomatch, pixel size and particle diameter were the only two parameters that were used to predict the particles. (./Gautomatch-{version} --apixM{pixel size} --diameter {particle diameter} /path of the folder containing micrographs).

### CASSPER performance

To test the performance of CASSPER, we selected four datasets that are commonly used for benchmarking, namely, HCN1^38^ (EMPIAR 10081), TRPV1^39^ (EMPIAR 10005), TcdA1^16,26^ (EMPIAR 10089), and β-galactosidase^40^ (EMPIAR 10017). Our selection includes proteins with different molecular weight (464 kDa–1.4 MDa) and proteins from different biological environments ranging from cytoplasmic to membrane proteins.

#### TcdA1

TcdA1 (EMPIAR 10089) is one of the well-studied components of tripartite ABC type toxin complexes released by nematodes in case of insect invasion. It has a molecular weight of 1.4 MDa, and its characteristic shape renders it distinguishable on the micrographs, making it a suitable candidate to develop an autopicking tool. This dataset has 97 movies which were acquired on Titan Krios with Falcon II detector (4k × 4k). The raw movies were motion-corrected by MotionCor2^41^, and CTF estimation was performed using CTFFIND4^42^ in RELION. Twenty-six micrographs were randomly picked and labeled for training via CASSPER. The actual training used 23 micrographs, and three micrographs were used for validation. The different validation parameters were monitored in all epochs. The SS model, with the highest F1 score, was used for making the predictions. The coordinates of the centers of the predicted particles were returned in box and star format. CASSPER showed the best performance for TcdA1 as it picked 11,245 good particles for the 3D reconstruction from 97 micrographs (Supplementary Table 1).

#### β-Galactosidase

β-Galactosidase (EMPIAR 10017) is a soluble protein that is routinely used for benchmarking cryo-EM data-processing tools and softwares. It is derived from *Escherichia coli* and forms a biological tetramer whose molecular weight corresponds to 464 kDa. The data were obtained from the EMPIAR database and were acquired by a POLARA microscope equipped with a Falcon II detector. For the study, 84 micrographs were used. Out of 44,261 particles picked from 84 micrographs by CASSPER, 40,467 were used for 3D reconstruction. Homogeneous refinement was performed by enforcing D2 symmetry for the 3D maps to obtain a resolution of 7.26 Å, which is better than the other tools under the uniform pipeline approach (Supplementary Table 1).

#### HCN1

HCN1 (EMPIAR 10081) is a membrane protein that plays a pivotal role in controlling the rhythmic activity of cardiac and neuronal cells. A total of 997 micrographs obtained from the EMPIAR database were used by all the three tools to predict the protein particles. After the 2D classification step in the uniform pipeline, 76% of the total number of particles picked by CASSPER were used for 3D reconstruction. The number of particles picked by the three tools for 3D reconstruction through the uniform pipeline and the corresponding resolutions are shown in Supplementary Table 1.

#### TRPV1

TRPV1 (EMPIAR 10005) is involved in mediating responses to various physical and chemical stimuli from the environment. For this dataset, 771 micrographs obtained from the EMPIAR database (collected on FEI POLARA 300 using GATAN K2 detector) were used to compare the performance of CASSPER with other tools through the uniform pipeline approach. Even though the total number of particles picked by CASSPER is less in this case, 2D class averages and 3D maps obtained were comparable with the other tools.

The 2D class averages for all the datasets obtained by processing the coordinates from different tools showed similar features (Supplementary Fig. 1). The EM density maps for β-galactosidase, TcdA1, TRPV1, and HCN1 shown in Supplementary Fig. 2a clearly indicate the difference in resolution achieved by these tools. Resolutions for all the EM density maps were estimated by Fourier shell correlation at FSC = 0.143 criterion indicated in Supplementary Fig. 2b–e.

### High-resolution 3D reconstruction

**Fig. 4:**
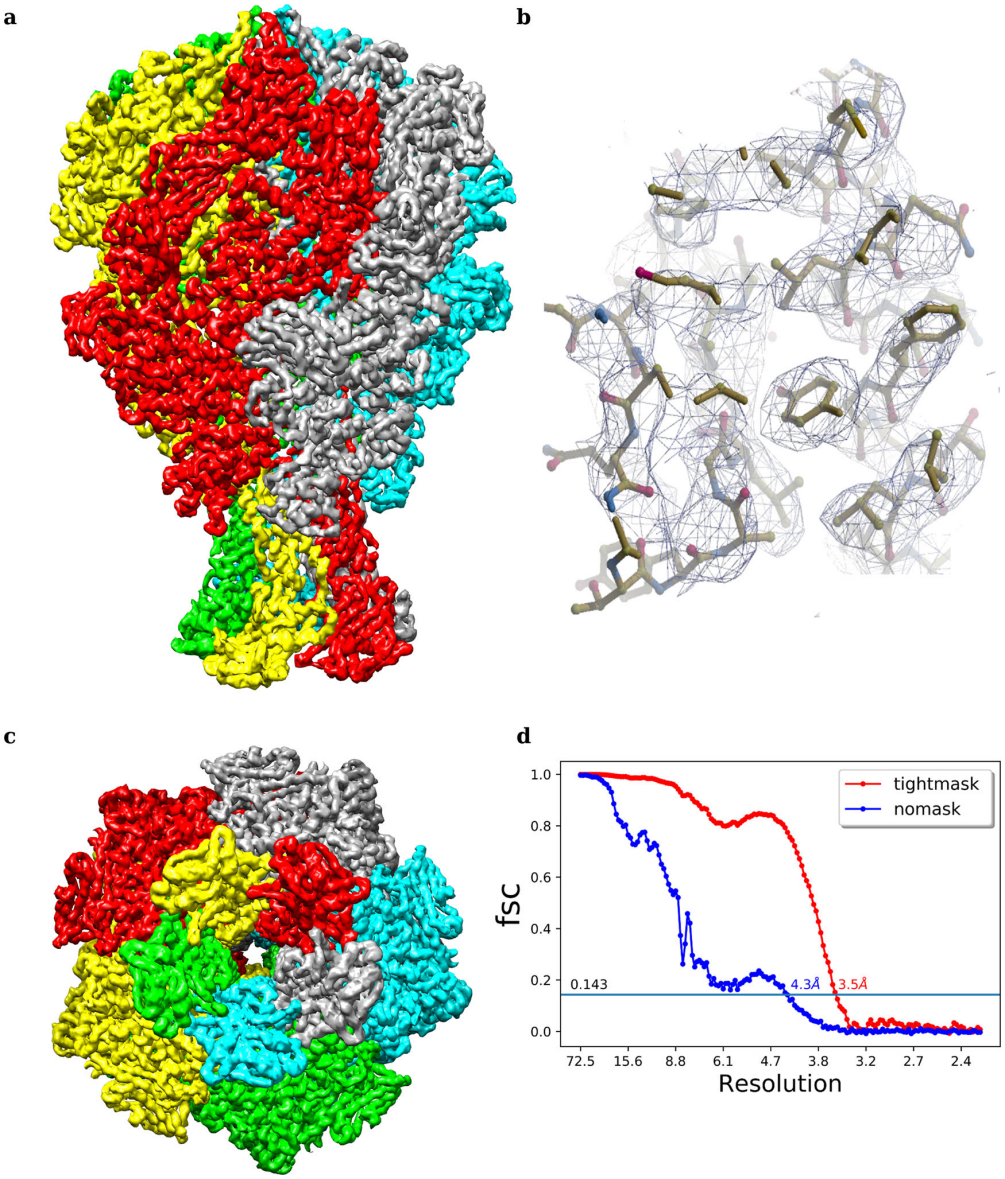
3D EM density map of TcdA1 obtained by implementing additional refinement steps to the uniform pipeline scheme. **a** Side view, **b** highlighted view of the side chains fitted (PDB 1VW1) into the EM density, **c** top view, and **d** FSC curve for TcdA1 showing resolution (Å) at gold standard cutoff (0.143).

**Fig. 5:**
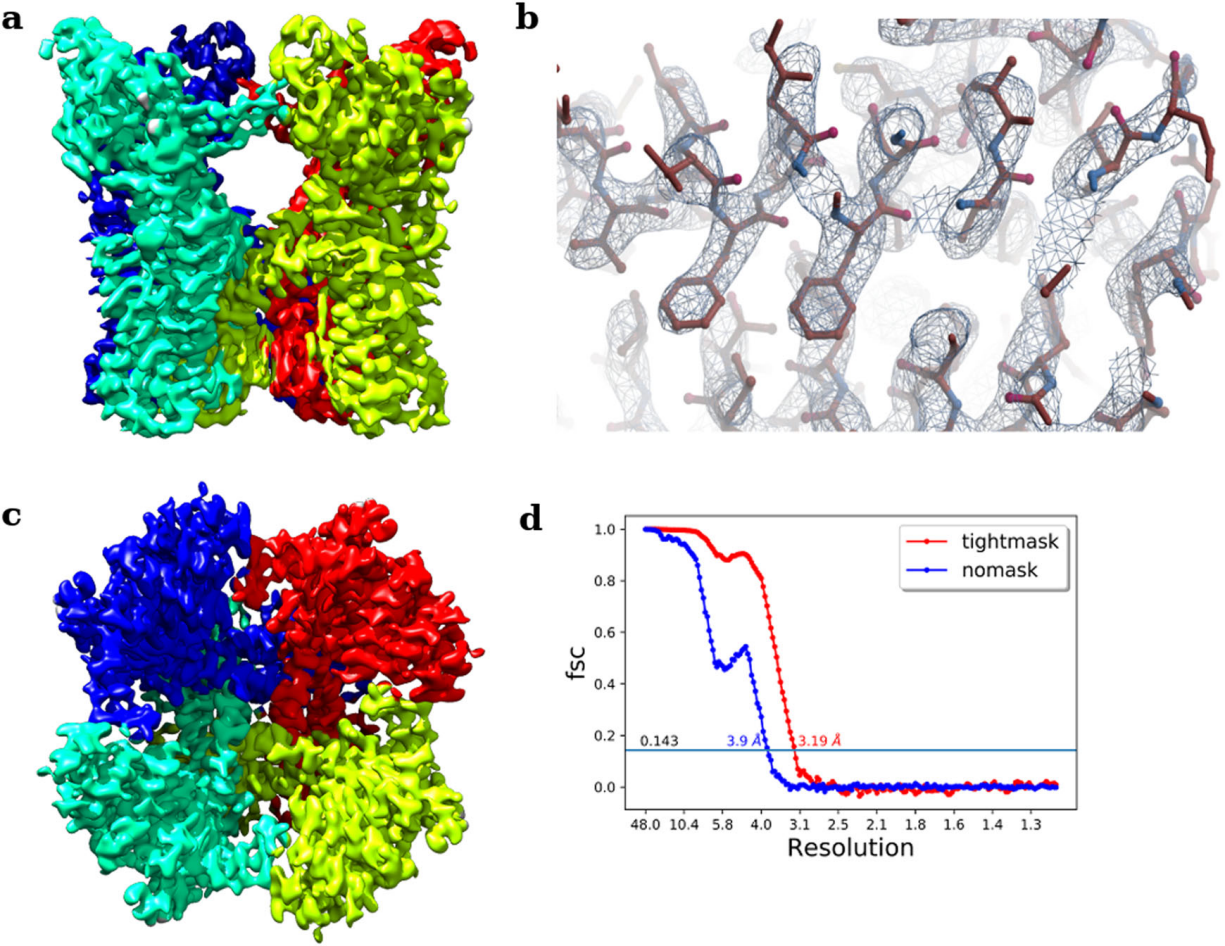
3D cryo-EM density of TRPV1 obtained by implementing additional refinement steps to the uniform pipeline scheme. **a** Side view, **b** highlighted view of the side chains fitted (PDB 3j5p) into the EM density, **c** top view, and **d** FSC curve for TRPV1 showing resolution (Å) at gold standard cutoff (0.143).

The ultimate goal of macromolecular structure determination is to explore biologically relevant intramolecular and intermolecular interactions in its native environment. This is possible only if we achieve a high-resolution structure which furnishes atomic-level details. To demonstrate the utility of CASSPER in obtaining high-resolution 3D reconstruction, we processed two (TcdA1 and TRPV1) of the four benchmarking datasets used in the uniform pipeline above. These two datasets were subjected to additional numbers of particle clean up by iterative 2D classification and 3D refinement steps like homogeneous, non-uniform, and local refinement protocols implemented in cryoSPARC v2 to achieve high-resolution 3D reconstructions. This additional processing yielded a resolution of 3.5 Å and 3.19 Å, for TcdA1 and TRPV1 respectively. Previous reports showed the resolution of TcdA1 with the same set of micrographs (EMPIAR 10089; EMD 3645) as 3.5 Å and TrpV1 structure was solved with same set of micrographs (EMPIAR 10005; EMD 5778) to achieve a resolution of 3.275 Å. Thus, resolution for TcdA1 obtained with CASSPER is equal to the published report; however, for TrpV1, the resolution achieved with CASSPER was better than that of the published reports^13,39^. Figures 4 and 5 show the final 3D maps of TcdA1 and TRPV1 obtained using the coordinates derived from CASSPER, where high-resolution features are clearly visible indicating high quality of EM density map obtained. This demonstrates the ability of CASSPER to automatically pick high-quality particles for high-resolution structure determination.

### Benchmarking CASSPER using the KLH dataset

Apart from the above benchmarking, we also used the standard KLH benchmark datasets^43^ to evaluate the performance of CASSPER. KLH dataset has micrographs imaged in pairs of high and low defocus allowing the evaluation of the effect of defocus on the picking efficiency. Using the track bars of our GUI, we labeled and trained CASSPER on 17 high defocus KLH micrographs. This trained model was used to pick particles on a separate set of 15 high defocus and 15 low defocus KLH micrographs. The Precision–Recall curves (Supplementary Fig. 3) gave an AUC of 0.96 and 0.915 for high and low defocus micrographs, respectively, which is comparable to that obtained by other tools (crYOLO^26^ and gempicker^19^) (Supplementary Table 2). This illustrates that the performance of CASSPER is at par with other tools and CASSPER has the ability to pick particles across variable defocus conditions. CASPPER being a SS-based method, detects MAVS filaments, stacked KLH particles as well but they are excluded due to particle radius defined during extraction of coordinates in the final step of prediction.

### Cross model performance of a generalized CASSPER model

**Fig. 6:**
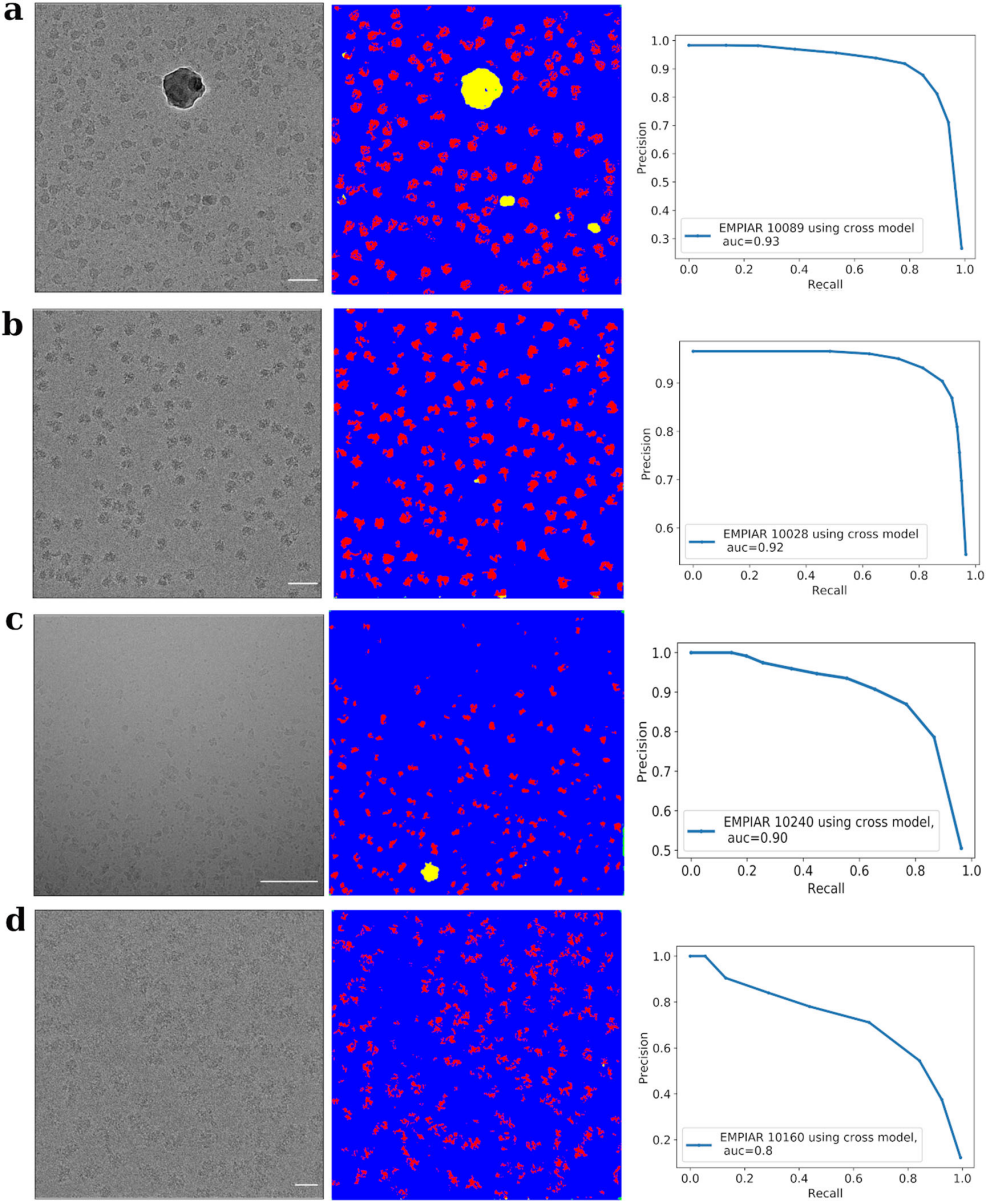
Evaluation of the performance of CASSPER cross model. Panels **a**–**d** show representative unlabeled micrograph (scale bar, 50 nm), semantically segmented and labeled micrograph using CASSPER cross model and AUC curves for proteins; **a** TcdA1 (EMPIAR 10089), **b** *Plasmodium falciparum* 80S ribosome bound to the anti-protozoan drug emetine (EMPIAR 10028), **c** afTMEM16/nanodisc complex (EMPIAR 10240), and **d** human Bact spliceosome (EMPIAR 10160), respectively. The high values of AUC score indicate the generalization ability of CASSPER. The PR curve is obtained by comparing the coordinates with manually picked ground truth labels.

Since CASSPER does not rely only on the morphological features, it has the ability to recognize proteins belonging to novel protein families with little information concerning their putative structures. In such cases, a generalized classifier or a pretrained model is required which can be used to predict particles on such unseen datasets. Hence, a generalized model was obtained by training CASSPER on 180 micrographs from 13 different protein datasets with a wide range of molecular weights (Supplementary Table 3). This general model was then used to predict a set of 15 randomly selected micrographs each of TcdA1 (EMPIAR 10089), 80S ribosome (EMPIAR 10028), afTMEM16/nanodisc complex (EMPIAR 10240), and Human Spliceosomal Bact Complex (EMPIAR 10160) that were not part of the training dataset. AUC scores were calculated as described in the “Methods” section and were 0.93, 0.92, 0.90, and 0.77 for EMPIAR 10089, EMPIAR 10028, EMPIAR 10240, and EMPIAR 10160, respectively. Representative micrographs for these datasets predicted by this general model and the precision–recall curves are shown in Fig. 6. The plots, with more than 90% scores, ascertain that our cross CASSPER model performs very efficiently in predicting the particles even for the unseen datasets, making it suitable for integration in any of the available cryo-EM data-processing pipelines.

### The SNR dependence

**Fig. 7:**
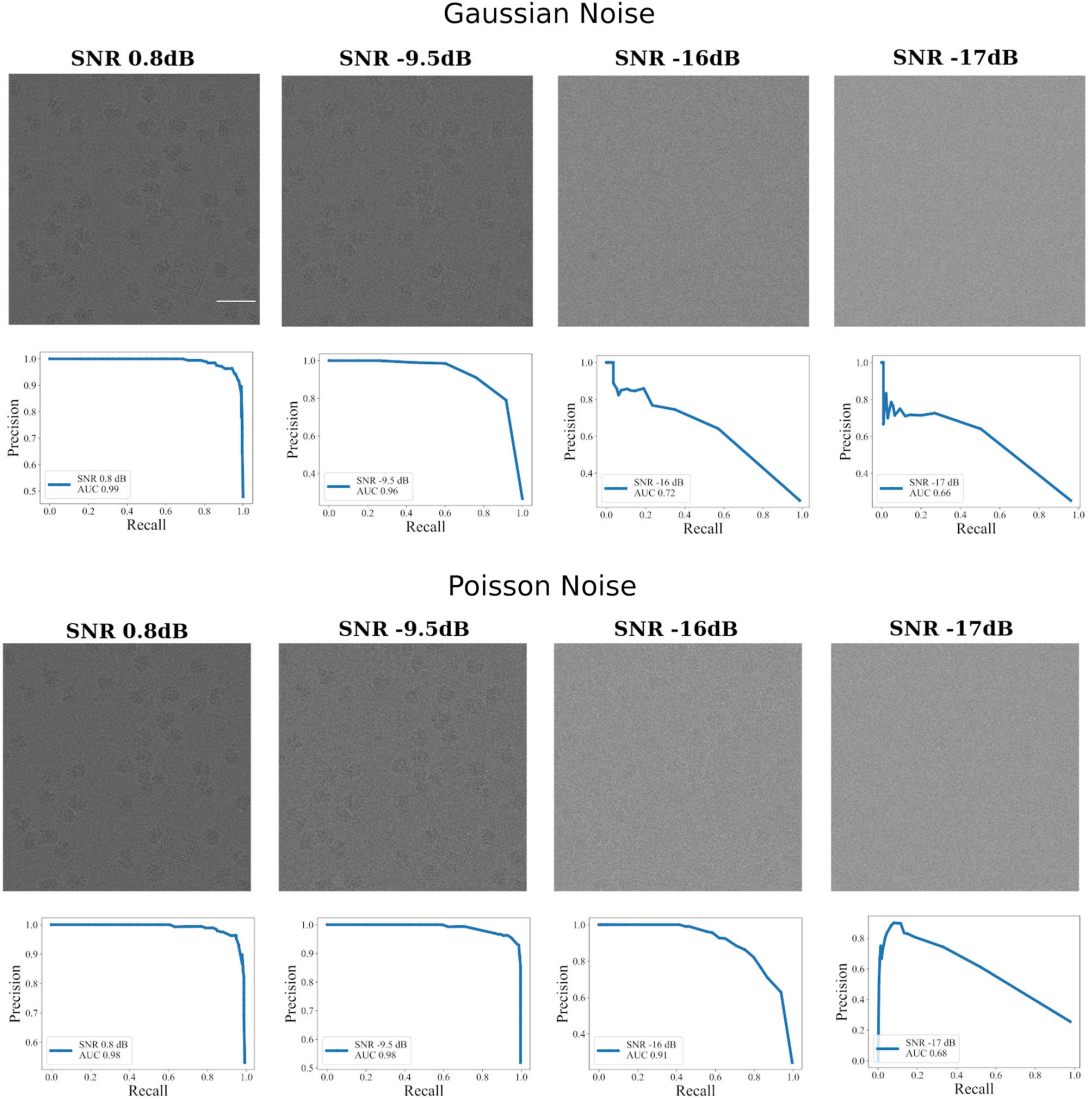
Analysis of the noise dependence on performance of CASSPER cross model. A representative EMPIAR 10028 (*Plasmodium falciparum* 80S ribosome bound to the anti-protozoan drug emetine) micrograph (scale bar, 50 nm) added with different levels of Gaussian and Poisson noise is shown along with the PR curve. The labels were predicted using CASSPER cross model.

One of the major reasons for reduced efficiency of automated particle picking tools is low SNR, therefore it is necessary to evaluate the performance of CASSPER over a range of SNRs. Different levels (0.8 dB–17.6 dB) of Gaussian and Poisson noise^44,45^ were introduced to the subset of 80S ribosome micrographs (EMPIAR ID 10028) (Fig. 7). The generalized CASSPER model was used to pick particles on each of the micrograph and it was observed that for Gaussian noise, the AUC and average precision (AP) were 0.96 and 0.88, respectively, up to a SNR of −9.5 dB (Fig. 7). The AUC was 0.93 up to a SNR of −15 dB (Table 1). Similarly, for micrographs with Poisson noise, performance of CASSPER was promising as the AUC and AP were found to be 0.91 and 0.88 or better up to a SNR of −16 dB (Fig. 7 and Table 1). These evaluations demonstrate robust performance of CASSPER in detecting particles even with low contrast and low SNR.

**Table 1:** The AUC and average precision scores for EMPIAR 10028 (*Plasmodium falciparum* 80S ribosome bound to the anti-protozoan drug emetine).

*Gaussian noise*								
SNR (dB)	0.8	−5.5	−7.5	−9.5	−11	−13	−16	−17.66
SNR	1.202	0.2828	0.1778	0.1122	0.0794	0.0501	0.0251	0.0171
AUC	0.991	0.985	0.981	0.964	0.932	0.893	0.725	0.660
Avg. precision	0.988	0.957	0.935	0.887	0.695	0.496	0.534	0.465
*Poisson noise*								
SNR (dB)	0.8	−5.5	−7.5	−9.5	−11	−13	−16	−17.66
SNR	1.202	0.2828	0.1778	0.1122	0.0794	0.0501	0.0251	0.0171
AUC	0.982	0.994	0.986	0.988	0.991	0.988	0.912	0.687
Avg. precision	0.980	0.990	0.983	0.983	0.988	0.984	0.881	0.495

The coordinates were predicted using CASSPER cross model. Different levels of Gaussian and Poisson noise were added to the micrograph and prediction. SNR values are shown in decibels as well.

### Computational efficiency and processing speed

A desktop computer with NVIDIA GeForce GTX 1070 graphics card, 64 GB RAM, and an Intel(R) Xeon (R) CPU was used to train CASSPER. With 12 micrographs for training for TcdA1 and KLH, CASSPER took 16 s for each epoch. The evaluation metrics are shown in Fig. 3a. With the same computer configuration, 0.94 s and 0.53 s were taken per micrograph to predict for a set of 97 TcdA1 and 82 KLH micrographs, respectively. This high processing speed suggests that CASSPER could be easily integrated into automated data-processing pipelines to pick particles while collecting data. Detailed comparison of processing speed of CASSPER with other tools has been summarized in Supplementary Table 4.

**Fig. 8:**
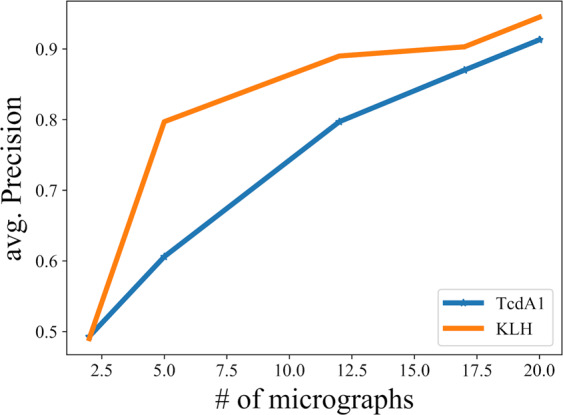
Effect of the number of micrographs used for training. Graph showing the average precision values for coordinates of TcdA1 and KLH which are predicted by models trained using different numbers of micrographs. It is to be noted that the number varies for different datasets. Training with at least 12 micrographs was required for KLH, whereas training with 20 micrographs was required for TcdA1 to get a prediction AUC score above 0.9.

The minimum number of micrographs required for training depends on the possible orientations, the shape of the protein, and the relative coverage of the particles in the micrographs. The relative coverage of KLH is 19%, and that of TcdA1 is 13%. Thus, it is observed that training with at least 12 micrographs was required for KLH, whereas training with 20 micrographs was required for TcdA1 to get a prediction AUC above 0.9 (Fig. 8).

## Discussion

In this study, we present a tool named CASSPER that can be used for automated particle picking from cryo-EM images. Using a powerful and robust SS deep learning framework, CASSPER assigns unique colors to pixels corresponding to various objects in micrographs such as ice, background, protein, and carbon edges, thereby labeling them as different classes. To the best of our knowledge, CASSPER is the first particle picking tool implementing the Residual Network architecture together with FRRN in pooling stream for efficient pixel-wise classification. Rather than searching for morphological features only, it takes into account the intrinsic differences in the scattering densities of protein and non-protein entities. Unique color labels are assigned to different classes such as protein, ice, and carbon based upon these variations. If a machine can learn how each pixel, corresponding to the protein particle, differs from non-protein entities, the collection of connected pixels can locate the position and shape of the protein. The particle coordinates are then extracted and returned in the popular box and star format for easy integration with any data-processing software package such as RELION. This method is only limited by the intrinsic differences in protein scattering density that may cause fragmentation in the label for a single protein structure. Since this can be corrected visually, we allow the user to specify a size threshold based on the labels predicted by CASSPER before it is used to count and pick individual particles.

In earlier segmentation-based tools like PIXER^27^, the feature map is segmented to get the protein-containing regions, and these regions are then given to a trained classifier to determine the center of particles. In CASSPER, the output of the SS network itself is classified into different classes in the micrograph, and no additional post-processing steps are needed for classification.

CASSPER offers a friendly GUI to allow the users to train the classifier on specific datasets without any manual particle picking. Apart from excellent performance of CASSPER model trained on specific datasets, CASSPER cross model also performs very well on unseen proteins. Efficiency of CASSPER as an automated particle picking tool remains consistent over a wide range of SNR. In addition, CASSPER cross model also performs promisingly on some low SNR datasets available in the lab (Supplementary Fig. 4), thus pointing at its robust performance.

## Methods

### Training of CASSPER

#### Training data preparation

**Fig. 9:**
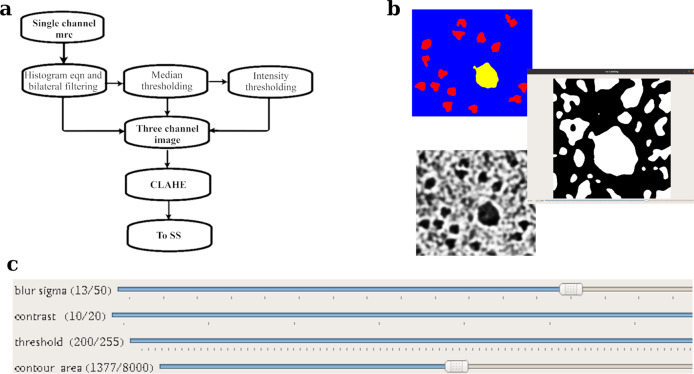
Flowchart representing pre-processing and labeling tool in CASSPER training phase. **a** The flowchart showing the pre-processing steps of CASSPER training phase. The single channel micrograph is enhanced and three different filters are applied to get the inputs for three channel images. **b** Figure showing the ice labeling method. **c** The toolbar section of the GUI which was used for labeling the proteins.

SS is a potent tool to learn the relation between an object and its surroundings. The semantically segmented labels required for training the network are determined using the CASSPER labeling tool. The processing steps of the tool are explained in Fig. 1. The raw micrograph image is enhanced using a Gaussian filter followed by contrast enhancement. The combination of range and domain filtering with bilateral filters preserves the image edges and removes the noise. In our implementation, the filter size is tuned using slide bars (Fig. 9). Sample vitrification usually leads to variable ice thickness in EM grid holes, which results in contrast differences in the micrographs and reduces the efficiency of particle picking tools. To address this limitation, we employed CLAHE^31^ on the images (Supplementary Fig. 5). The contrast limiting (CL) value for CLAHE can be adjusted using the slide bar. Usually, low positive CL values are suitable for most proteins. The final segmentation in the enhanced image is done in two steps: (a) intensity thresholding and (b) size filtering. Track bars are used to set the threshold values for the intensity and the area of the particles. Based on these inputs, the final labels are created. This is a one-time procedure performed on a training subset, and the generated values can be used for prediction on all the micrographs of the dataset under consideration, thereby making the labeling procedure extremely fast and accurate. In addition to protein particles, by following the same procedure, ice and carbon contaminants that have different scattering density are also labeled with different colors.

#### Pre-processing

The grayscale cryo-EM images have very low SNR, so they have to be enhanced before submitting to the SS algorithm. Also, the SS algorithm can work on multichannel color images and in our implementation, the three-channel input images are obtained by applying three different filters to the motion-corrected micrographs. In the first channel, contrast enhancement and edge preserved noise removal of the input image is done by histogram equalization followed by bilateral filtering. The second channel is prepared by median thresholding that retains only pixels with intensities around a set threshold of the median pixel. In effect, this channel enhances the contrast around the median pixel range of the image. The third channel is generated by applying a Gaussian adaptive threshold to the second channel. These enhanced images are then combined to form a three-channel image. This image is then adaptively histogram equalized using CLAHE to reduce the contrast difference effects and improve the efficiency of SS.

#### Test for precision–recall curve

Published particle annotations were used as ground truth to evaluate the quality of the predicted labels. Manually annotated labels by experts were used in case of datasets where particle annotations were unavailable. However, in SS, each pixel is assigned a probability to be a member of one of the classes. A cluster of pixels of the same kind that has more than some threshold representation is labeled as the location of the particle. The PR curve is plotted by varying this threshold, and the value corresponding to the maximum F1 score is taken as the final threshold for prediction.

### Statistics and reproducibility

Definition of the statistical terms used.

*True positive*: Number of pixels that are predicted to the correct class.

*False positive*: Number of pixels that are predicted to a wrong class.

*Precision*: Percentage of correct predictions.

*Recall*: Ratio of correct pixels in the predicted label to the ground truth.

*Intersection over Union (IoU)*: Ratio of the number of common pixels in the predicted and ground truth images to the union of the pixels in both images.

*F1 score*: Weighted average of precision and recall,

$${\mathrm{F}}_1\,{\mathrm{Score}} = 2\,\times\left( {{\mathrm{precision}}\,\times\,{\mathrm{recall}}} \right)/({\mathrm{precision}} + {\mathrm{recall}})$$

is used as the parameter for evaluating the validation performance, and the model with the highest F1 score is used for the prediction of the unseen proteins.

*Average precision*: AP summarizes PR curve as the weighted mean of precisions achieved at each threshold value. The increase in recall from the previous threshold is used as the weight AP = Σ_*n*_(*R*_*n*_ − *R*_*n−*1_)*P*_*n*_, where *R*_*n*_ and *P*_*n*_ are the recall and precision at the *n*th threshold^46^.

For evaluating the performance of the prediction using the cross model, we employed F1, accuracy, and mean IoU scores to pixel-wise compare the predicted labels with the labels made using our labeling tool. The weighted average of particle and non-particle pixels are indicated in these scores.

### Reporting summary

Further information on research design is available in the Nature Research Reporting Summary linked to this article.

## Supplementary information

Peer Review File

Supplementary Information

Description of additional supplementary files

Supplementary Data 1

Reporting Summary

## Data Availability

The training datasets for this study, particle stacks, and 2D class stacks are available on the GitHub page “CASSPER” along with a detailed practical manual for download under the GitHub page https://github.com/airis4d/CASSPER.

## Code availability

The source code is contained in the CASSPER software package which is available at GitHub page (https://github.com/airis4d/CASSPER) and Zenodo (10.5281/zenodo.4289389). Its use is restricted by the end-user license agreement: Creative Commons License.
