# Supplementary material for: CASSPER is a semantic segmentation-based particle picking algorithm for single-particle cryo-electron microscopy
Source: Commun Biol. 2021 Feb 15;4:200. doi: 10.1038/s42003-021-01721-1 (PMC7884729; doi:10.1038/s42003-021-01721-1)
Supplement: Supplementary file 2 — Supplementary Information [file 42003_2021_1721_MOESM2_ESM.pdf]

CASSPER is A Semantic Segmentation-based Particle Picking Algorithm for Single-Particle Cryo-Electron Microscopy

Blesson George [1,5,‡], Anshul Assaiya[2,‡], Robin J. Roy[1], Ajit Kembhavi[3], Radha Chauhan[4], Geetha Paul[1], Janesh Kumar[2,*], Ninan S. Philip[1,*]

[1]Artificial Intelligence Research and Intelligent Systems (airis4D), Thelliyoor - 689544, Kerala, India

[2]Laboratory of Membrane Protein Biology, National Centre for Cell Science, NCCS Complex, S. P. Pune University Campus, Ganeshkhind, Pune- 411 007, INDIA

[3]Inter-University Centre for Astronomy and Astrophysics (IUCAA), S. P. Pune University Campus, Ganeshkhind, Pune- 411 007, INDIA

[4]Laboratory of Structural Biology, National Centre for Cell Science, NCCS Complex, S. P. Pune University Campus, Ganeshkhind, Pune- 411 007, INDIA

[5] Department of Physics, CMS College, Kottayam - 686001, Kerala, INDIA

[‡] **Equal contribution**

Supplementary Information

***Corresponding authors**

Ninan Sajeeth Philip PhD
Dean and Director,
Artificial Intelligence Research and Intelligent Systems (airis4d.com)
Thelliyoor -689644
Kerala, INDIA
Email: ninansajeethphilip@gmail.com

Janesh Kumar, Ph.D.
Scientist E,
National Centre for Cell Science
NCCS Complex, Pune University Campus
Ganeshkhind, PUNE- 411 007, INDIA
Email: janesh@nccs.res.in

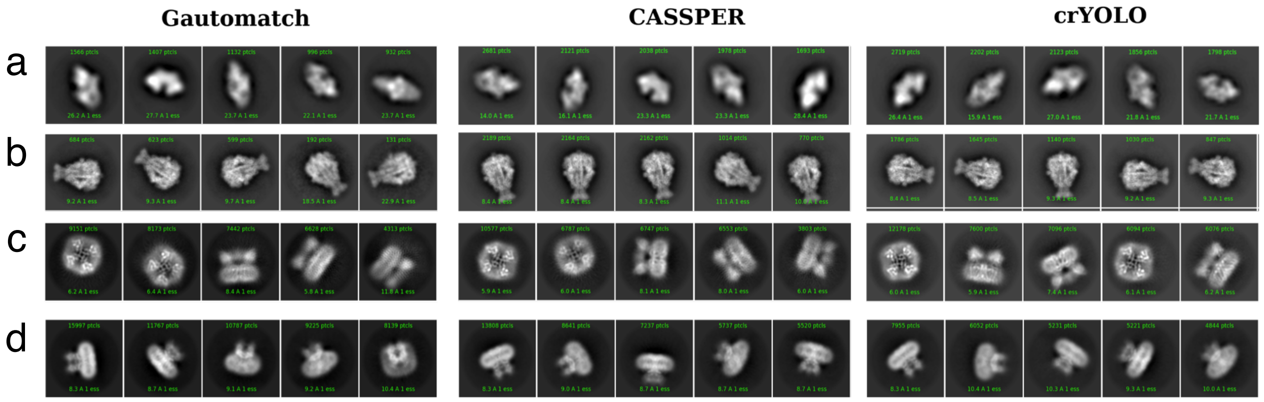

Supplementary Figure 1. Comparison of Representative 2D class averages. a β-galactosidase, **b** TcdA1, **c** TRPV1 and **d** HCN1 obtained after a single round of 2D classification in uniform pipeline using the particles picked by different tools.

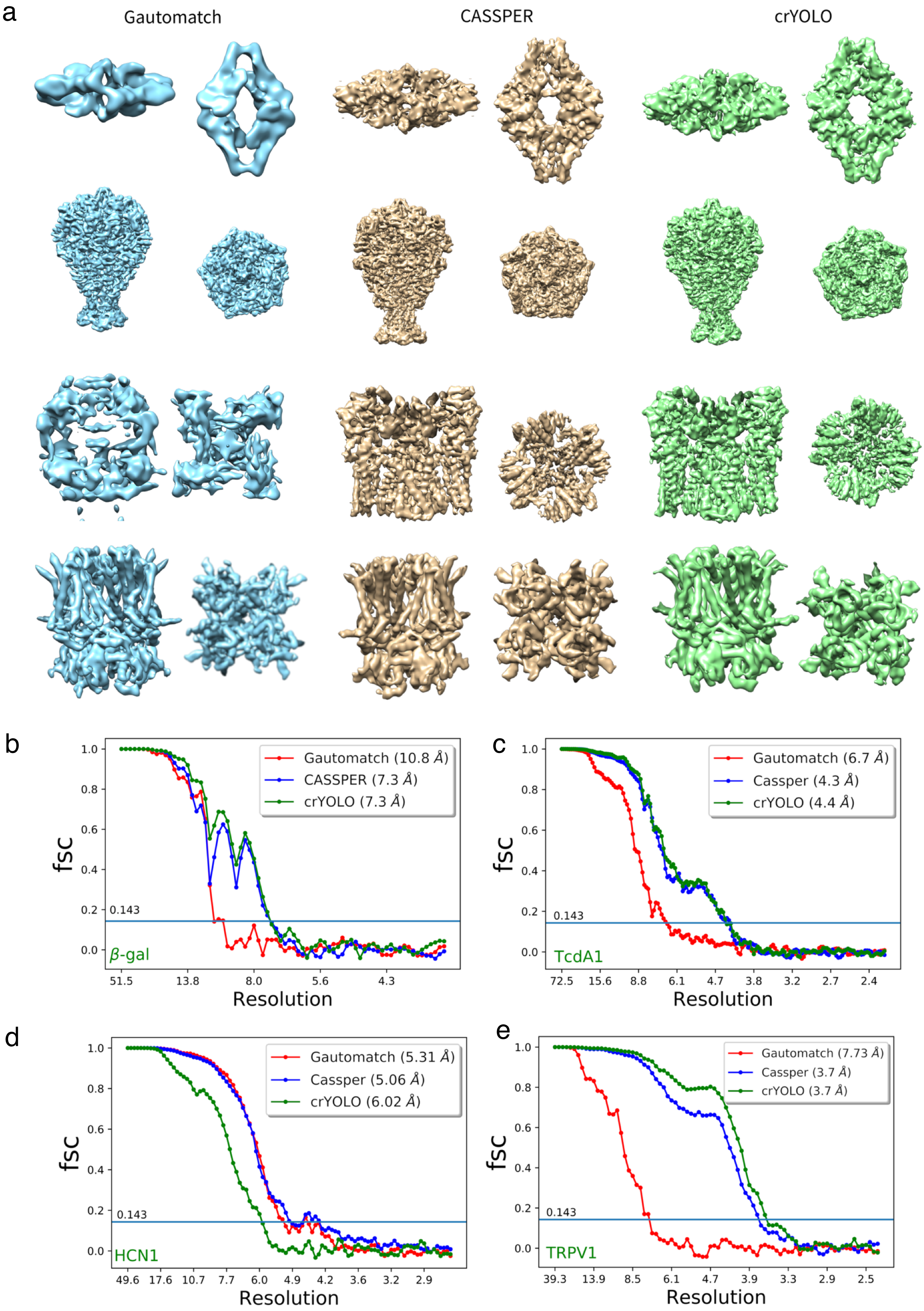
a
Gautomatch
CASSPER
crYOLO
b
Gautomatch (10.8 Å)
CASSPER (7.3 Å)
crYOLO (7.3 Å)
fsc
0.143
β-gal
Resolution
c
Gautomatch (6.7 Å)
Cassper (4.3 Å)
crYOLO (4.4 Å)
fsc
0.143
TcdA1
Resolution
d
Gautomatch (5.31 Å)
Cassper (5.06 Å)
crYOLO (6.02 Å)
fsc
0.143
HCN1
Resolution
e
Gautomatch (7.73 Å)
Cassper (3.7 Å)
crYOLO (3.7 Å)
fsc
0.143
TRPV1
Resolution

Supplementary Figure 2. Evaluation of CASSPER using uniform pipeline. Comparison of 3D models for β-galactosidase, TcdA1, TRPV1 and HCN1 generated using particles picked by Gautomatch (blue), CASSPER (tan), crYOLO (green)**.** The particles were extracted in RELION2 and further processing was done using cryoSPARC v1 as per the uniform pipeline scheme. **a** Different views of the 3D models generated for β-galactosidase, TcdA1, TRPV1, and HCN1. FSC curves (tight mask) for the 3D reconstruction of β-galactosidase **(b)**, TcdA1 **(c)**, TRPV1 **(d)** and HCN1 **(e)** showing the resolution at the gold standard cut off (0.143) obtained using Gautomatch (red), CASSPER (blue) and crYOLO (green).

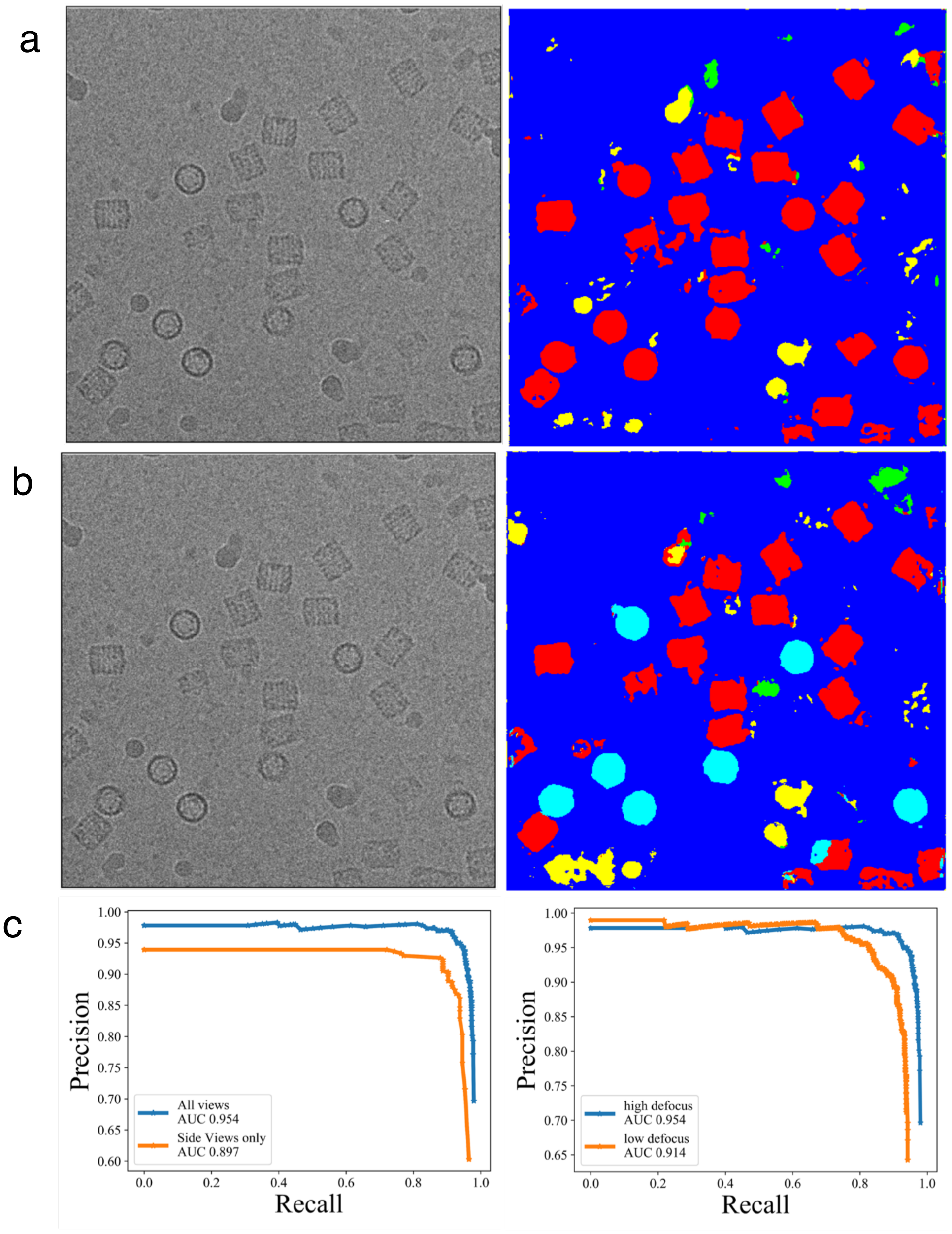

Supplementary Figure 3. CASSPER benchmarking using KLH dataset. Particles picked by CASSPER (**a**) all views (**b**) side views on representative micrograph of KLH and Precision recall curves. Pixels labelled in red, blue, green and yellow color correspond to protein, background, carbon edges and ice/liquid ethane respectively. **c** Precision recall curves for depicting performance of CASSPER for picking all views, only side views and for low and high defocus micrographs.

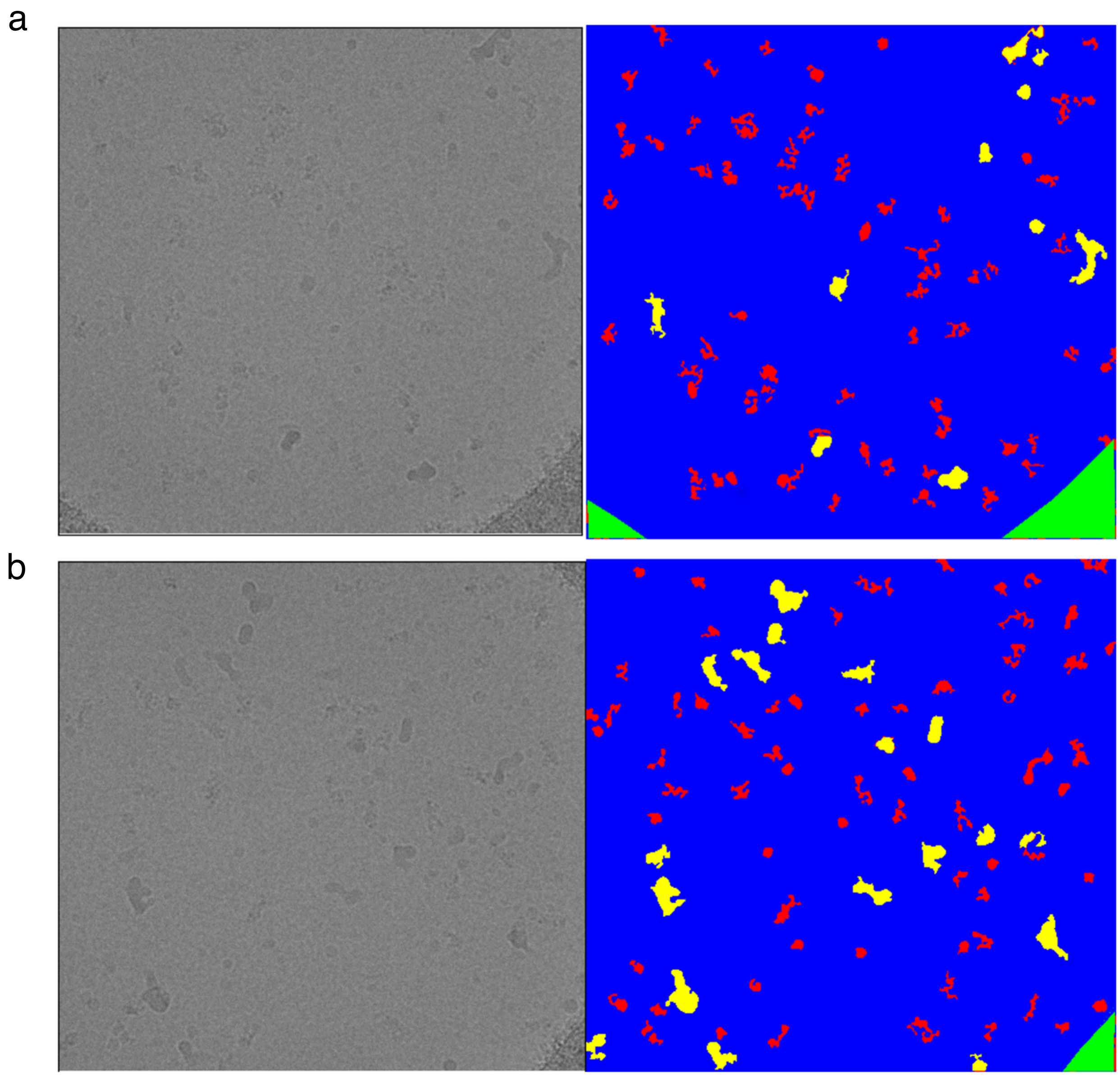

Supplementary Figure 4. CASSPER performance on GluK3 receptor micrographs. Representative micrographs of GluK3-kainate receptors (**a**) and (**b**) showing particles selected by CASSPER general model. Carbon edges are labelled in green, background in blue, ice in yellow and protein in red.

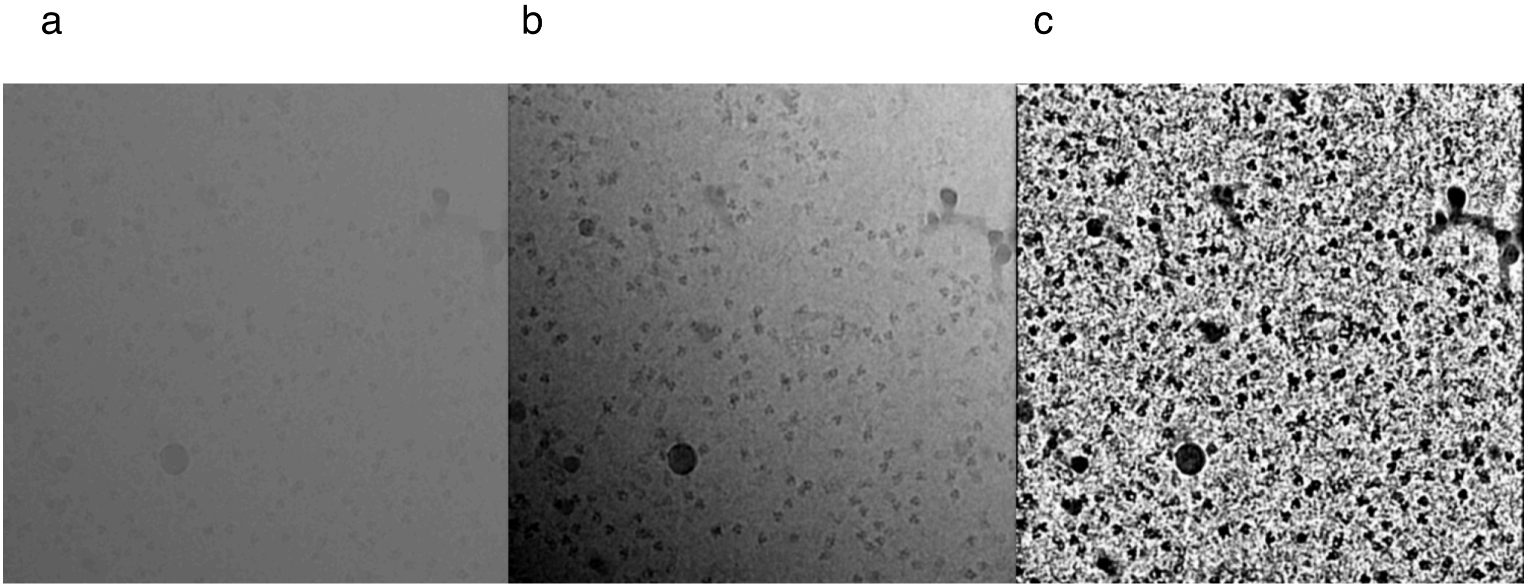

Supplementary Figure 5. Effect of CLAHE. **a** The raw micrograph, **b** The image is enhanced without applying CLAHE. The contrast difference at different regions of the image can be noted. **c** The image is enhanced after applying CLAHE. It can be observed that CLAHE has removed the contrast difference in the image.

Supplementary Table 1. Comparison of Gautomatch, CASSPER and crYOLO for β-galactosidase, TcdA1, TRPV1 and HCN1. The total number of particles picked by the respective tools were fed into the uniform pipeline scheme for further processing. The 2D class averages with characteristic features were selected and used for 3D reconstruction followed by homogeneous refinement for all proteins by imposing the respective symmetry. The resolutions obtained through a uniform pipeline scheme are given in the table.

Protein	Method	No of particles picked	No of particles selected for 3D reconstruction	Resolution (Å)
**TcdA1** EMPIAR 10089	GAUTOMATCH	4097	3364	6.7
	CASSPER	14603	11245	**4.3**
	crYOLO	11127	10629	4.4
**β-gal** EMPIAR 10017	GAUTOMATCH	25409	21476	10.8
	CASSPER	44261	40467	**7.26**
	crYOLO	44591	42876	7.32
**HCN1** EMPIAR 10081	GAUTOMATCH	195782	107332	5.31
	CASSPER	150342	115297	**5.06**
	crYOLO	141002	103010	6.02
**TRPV1** EMPIAR 10005	GAUTOMATCH	127776	38836	7.73
	CASSPER	107320	46913	3.74
	crYOLO	110153	67269	**3.68**

Supplementary Table 2. Precision recall curves showing the performance of CASSPER generalized model on KLH micrographs at high and low defocus values as per the bake off criterion.

**Model trained using 17 high defocus micrographs**			
**Predicted for 15 micrographs**	**AUC**	**Precision**	**Recall**
High defocus	0.954	0.944	0.948
Low defocus	0.92	0.90	0.898

Supplementary Table 3: List of datasets used for training general model of CASSPER

Sr. No.	EMPIAR ID	Protein Name
1	10272	Horse spleen apoferritin
2	10025	T20s Proteasome
3	10096	Influenza Hemagglutinin Trimer
4	10175	Hemagglutinin
5	10215	Rabbit muscle aldolase
6	10217	Bovine liver glutamate dehydrogenase
7	10285	P-Rex-1-G-beta gamma signaling factor
8	10168	RNA Polymerase III
9	10208	Mouse MDA5-dsRNA
10	10081	(human HCN1 hyperpolarization-activated cyclic nucleotide-gated ion channel)
11	10005	TRPV1
12	10099	Hrd1 and Hrd3 complex
13	JSB,Vol.145, pp. 3-14,2004)	KLH dataset

Supplementary Table 4: Comparison of processing speed tested on the same set of 15 micrographs each for TcdA1, TrpV1, β-gal and KLH using CASSPER, crYOLO and Topaz. Parameters such as downscaling factor, particle radius, and size of training dataset were kept uniform for each datasets when tested with different tools and the experiment was performed on the same desktop employing one GPU.

Protein	CASSPER (seconds/mrc)	crYOLO (seconds/mrc)	Topaz (seconds/mrc)
**TcdA1**	1.92	1.87	1.87
**TRPV1**	1.76	2.23	1.5
**β-gal**	1.8	2.8	1.89
**KLH**	1.2	1.66	0.85
