# Supplementary material for: CASSPER is a semantic segmentation-based particle picking algorithm for single-particle cryo-electron microscopy
Source: Commun Biol. 2021 Feb 15;4:200. doi: 10.1038/s42003-021-01721-1 (PMC7884729; doi:10.1038/s42003-021-01721-1)
Supplement: Supplementary file 5 — Reporting Summary [file 42003_2021_1721_MOESM5_ESM.pdf]

nature research

Corresponding author(s): Janesh Kumar

Last updated by author(s): Dec 2, 2020

Reporting Summary

Nature Research wishes to improve the reproducibility of the work that we publish. This form provides structure for consistency and transparency in reporting. For further information on Nature Research policies, see Authors & Referees and the Editorial Policy Checklist .

Statistics

For all statistical analyses, confirm that the following items are present in the figure legend, table legend, main text, or Methods section.

n/a	Confirmed	
☐	☒	The exact sample size (*n*) for each experimental group/condition, given as a discrete number and unit of measurement
☒	☐	A statement on whether measurements were taken from distinct samples or whether the same sample was measured repeatedly
☒	☐	The statistical test(s) used AND whether they are one- or two-sided<br>*Only common tests should be described solely by name; describe more complex techniques in the Methods section.*
☒	☐	A description of all covariates tested
☐	☒	A description of any assumptions or corrections, such as tests of normality and adjustment for multiple comparisons
☐	☒	A full description of the statistical parameters including central tendency (e.g. means) or other basic estimates (e.g. regression coefficient) AND variation (e.g. standard deviation) or associated estimates of uncertainty (e.g. confidence intervals)
☒	☐	For null hypothesis testing, the test statistic (e.g. *F*, *t*, *r*) with confidence intervals, effect sizes, degrees of freedom and *P* value noted<br>*Give P values as exact values whenever suitable.*
☒	☐	For Bayesian analysis, information on the choice of priors and Markov chain Monte Carlo settings
☒	☐	For hierarchical and complex designs, identification of the appropriate level for tests and full reporting of outcomes
☒	☐	Estimates of effect sizes (e.g. Cohen's *d*, Pearson's *r*), indicating how they were calculated

Our web collection on statistics for biologists contains articles on many of the points above.

Software and code

Policy information about availability of computer code

Data collection	Data used in this study were downloaded from the EMPIAR database. We also included inhouse data which were collected on a Titan Krios..<br>GitHub page https://github.com/airis4d/CASSPER and Zenodo (doi:10.5281/zenodo.4289389)
Data analysis	GitHub page https://github.com/airis4d/CASSPER .

For manuscripts utilizing custom algorithms or software that are central to the research but not yet described in published literature, software must be made available to editors/reviewers. We strongly encourage code deposition in a community repository (e.g. GitHub). See the Nature Research guidelines for submitting code & software for further information.

Data

Policy information about availability of data

All manuscripts must include a data availability statement. This statement should provide the following information, where applicable:

- Accession codes, unique identifiers, or web links for publicly available datasets
- A list of figures that have associated raw data
- A description of any restrictions on data availability

The training datasets for this study, particle stacks, and 2D class stacks are available on the GitHub page “CASSPER” along with a detailed practical manual for download under the GitHub page https://github.com/airis4d/CASSPER . Software code also available at Zenodo (doi:10.5281/zenodo.4289389)

Field-specific reporting

Please select the one below that is the best fit for your research. If you are not sure, read the appropriate sections before making your selection.

Life sciences study design

All studies must disclose on these points even when the disclosure is negative.

Sample size	The number of micrographs for training or picking particles were kept constant for all the tools.
Data exclusions	No data were excluded from the analysis.
Replication	Training Epoch, particle picking efficiency were reproducible with our training data available at the github page. However, with different training data the results might be slightly different.
Randomization	Micrographs were randomly chosen for training set and for particle picking. The micrographs in the training set and picking (test set) were unique and non-overlapping.
Blinding	Not applicable

Reporting for specific materials, systems and methods

We require information from authors about some types of materials, experimental systems and methods used in many studies. Here, indicate whether each material, system or method listed is relevant to your study. If you are not sure if a list item applies to your research, read the appropriate section before selecting a response.

Materials & experimental systems

n/a	Involved in the study	
☒	☐	Antibodies
☒	☐	Eukaryotic cell lines
☒	☐	Palaeontology
☒	☐	Animals and other organisms
☒	☐	Human research participants
☒	☐	Clinical data

Methods

n/a	Involved in the study	
☒	☐	ChIP-seq
☒	☐	Flow cytometry
☒	☐	MRI-based neuroimaging
