# Supplementary material for: CASSPER is a semantic segmentation-based particle picking algorithm for single-particle cryo-electron microscopy
Source: Commun Biol. 2021 Feb 15;4:200. doi: 10.1038/s42003-021-01721-1 (PMC7884729; doi:10.1038/s42003-021-01721-1)
Supplement: Supplementary file 1 — Peer Review File [file 42003_2021_1721_MOESM1_ESM.pdf]

Reviewers' comments:

Reviewer #1 (Remarks to the Author):

The manuscript by George et al presents a new method named CASSPER, which applies a deep learning-based sematic segmentation for automated particle picking and selection from EM micrographs. The authors tested their method using a number of experimental datasets and demonstrate certain improvement and potential advantage over existing methods. The results were presented in great detail, which appear to support the applicability of the proposed method, at least in the form of what is presented. This could be a welcoming contribution for the field of cryo-EM methods development. However, several questions should be addressed before the manuscript can be accepted for publication.

(1) There is a lack of a baseline characterization of their method, and a baseline benchmark of the performance that can be used for comparison with existing methods. There is a consensus "bake-off" dataset (the KLH dataset available through http://emg.nysbc.org/redmine/projects/public-datasets/wiki/KLH_datasets) that has been tested for most particle picking algorithms developed so far. Providing testing results on this standard dataset presents a baseline benchmark that can be readily used for comparison with other methods in literature.
(2) The authors should present the precision-recall curves of their tests, particularly using the consensus "bake-out" KLH dataset. Otherwise, it is hard to know to what degree the implementation improves over a large number of existing methods. The comparison of final 3D maps among selected methods is useful but it is also affected by other steps in data processing, making it hard to know the net performance difference in particle picking step.
(3) How many particles are needed for training data? What is the lower and higher bound of the size of training dataset?
(4) How is the approach performed compared to another deep-learning-based particle picking method TOPAZ (Nature Methods 16, 1153, 2019)?
(5) The grey scale of raw micrographs in Figure are not properly presented. It is either too bright or too dark.
(6) The Methods section includes insufficient details to assess the soundness of the tests and thus validity of the method. Can the authors provide the precision-recall curves for their tests on the generalized model for cross-molecule picking as well as details about the tests?
(7) How efficient is the authors' implementation? How is their computational efficiency compared to other methods?
(8) How does this method behave over a large range of SNRs due to variation in particle size and defocus? What is the lowest SNRs that the method can afford?
(9) On page 5, the authors claim that "CASSPER is the first method to carry out labelling and prediction of different kinds of particles in a micrograph". This may not be true. Even the earliest computational method in particle picking has to have some ability in labelling and prediction of different kinds of particles. The only difference is the precision and accuracy. I suggest that the authors revised this sentence to "CASSPER is the first Sematic Segmentation-based method to … in a EM micrograph".
(10) In the introduction, the authors described Gautomatch, which was based on FindEM, as "template-free methods". This is wrong. FindEM/Gautomatch was one of the early methods based on template matching algorithm.

Reviewer #2 (Remarks to the Author):

George et al present a deep-learning based program for automatic particle picking in electron micrographs. They argue the need for better programs and compare the performance of their package, named CASSPER, with two other programs used in the field (gautomatch and cryolo) in terms of number of particles found within a (subset of) 4 empiar datasets as well as the resolution

of a 3d reconstruction obtained from a subset of these particles. Table 1 provides the numbers for this comparison. In bold, the authors highlight that the best resolution was obtained, for three out of the four data sets, when using CASSPER for initial particle picking.

Their program uses a pixel-level classification and can produce coloured micrograph representations in which ice, protein, and carbon are classified differently (figure 2). Figure 3C shows the picking result of the three different programs for an individual micrograph from the each of the four data sets. In green, areas have been highlighted where the other two progs pick up false positives, whereas CASSPER correctly did not pick particles.

The introduction contains a number of claims highlighting the need for a new particle picking scheme, such as "One of the obstacles that still remains unresolved", "picking, and selection is a very tedious and challenging process", "laborious, slow, manual or semiautomated methods", "However, these tools are prone to picking huge amount of contaminants, background, and ice, and do not work optimally", "manual particle picking usually introduces a strong template bias". I must have manually picked tens to hundreds of thousands of particles in my career, and although that was sometimes laborious indeed, it was not necessarily slow within a wider scheme of solving a novel structure. Contaminations, ice and background can be taken care of already (by adjusting data collection schemes, picking parameters, templates, and 2d classification), and template bias can also be used to own's advantage if a micrograph contains different particles that one wish to separate as early as possible. It occurred not seldom to me that people who I introduced new into cryo-EM, directly wished to write a new program to improve on the particle picking process. Personally, I am not convinced that that is the biggest challenges within our field that we need to overcome right now.

Their most convincing argument is, imo, "A fast, automatic method that can replace the manual processing is thus a necessity for automating the structure determination process." Smart microscopy would require direct feedback of the premise of the data while it is being collected. Automated particle picking would indeed be mandatory for this. Unfortunately, the manuscript does not comment on the processing speed of CASSPER. Can it keep up with automated data collection? Within the manuscript, the prog has only been tested on empiar data sets. However, most data sets don't end up in empiar, for a wide variety of reasons. Will it also perform well for real-life data, of which only such a small percentage proved to be good enough for deposition in empiar? This could, for example, be evaluated by running CASSPER real-time within a cryo-EM center for a while, maybe even at ACCEM-IISc?

The authors overlooked the Nature Methods paper of Tegunov and Cramer, which was published on october 07 2019 but has been in biorxiv since jun 14 2018. It deals with real time cryo-EM processing, and does include a novel deep-learning based particle picking scheme already, named BoxNet, which is based on residual network as implemented in TensorFlow. "CASSPER uses the SS implementation by George Seif" which is also a suite in TensorFlow.

Specific comments
1. How does CASSPER perform compared to BoxNet?
2. What are the differences between the two methods and why are these differences important?
3. Could CASSPER be used for real-time cryo-EM particle picking?
4. A 80s ribosome is not a protein
5. The authors write "The ultimate goal of macromolecular structure determination is to explore biologically relevant intramolecular and intermolecular interactions in its native environment." Could CASSPER also be used for picking particles in tomograms of macromolecular complexes within their native, cellular environment?
6. Surely, it should be possible to find more particles in TcdA1 using gautomatch (table 1)?
7. Idem for beta-gal
8. Is the resolution of a final 3d reconstruction the best criterium to evaluate CASSPER?

Reviewer #3 (Remarks to the Author):

This paper introduces a new method for particle picking based on deep learning. I am unaware of another paper that suggests the use of a full-resolution residual network. This method is shown to achieve results comparable to state-of-the-art methods on two datasets, and provides a nice experiment showing generalization. I believe that this paper will be of interest to the cryo-electron microscopy community. However, I do have some questions about this method and its general applicability.

My main concern is that the authors claim it is vital to accurately label each pixel in the training set. This is known to be difficult (if not impossible). The high levels of noise present in the micrograph cause edge-detection-based methods to fail. Furthermore, the contrast transfer function causes the projection to seem to exist beyond its edges. How are these issues dealt with?

Additionally, I have some questions about the results:

1. In the evaluation the authors explain that "12 micrographs that do not constitute the training dataset were randomly selected… and mean values of F1, IoU and accuracy scores… were obtained by comparing predicted labels with the corresponding ground truth". How were the ground truth values obtained?

2. Some of the experiments achieve rather bad resolutions (for example, the EMPIAR 10017 dataset which is often used to test methods of particle picking). Can these results be explained?

Other questions I feel should be answered in the manuscript are:

Introduction:

3. The authors claim that "the exposure differences, noise levels and variable ice thickness in micrographs also limits the performance of these tools". First, it was unclear to me which tools are meant in this sentence. Second, it is unclear to me what this claim is based on. Lastly, what effect do these factors have on the suggested particle picker?

4. The authors claim that CASSPER uses the "transmittance of the medium". What is meant by transmittance? The scattering density?

5. The authors claim that CLAHE is used for particle identification. Indeed, this is the goal of the entire paper. What is the specific benefit of using CLAHE? (e.g. as opposed to other histogram equalization methods).

6. I think it should be made clear in the section that there is no manual picking involved as that is a rather large advantage of this method.

Implementation of CASSPER

7. The explanation about FRRNs is unclear and should be rewritten. For example, the function f(yn-1;wn) is used twice, once for the feed-forward network and once for the residual network. Is this the same function? Also, how is f related to g and h? and how is y related to j and k?

"Uniform pipeline for comparison":

8. By CTF-estimated micrographs, do the authors mean CTF corrected?

High-resolution 3D reconstruction

9. The resolution of TRPV1 and TcdA1 published in EMDB should be added.

10. It is unclear to me why the authors report two different resolutions for TRPV1 and TcdA1 since both reconstructions were achieved from the same picked particles. That is, since cryoSPARK produces the best results in the experiment, why report the resolution achieved by a different method in the first place? also, why weren't the molecules reconstructed from the crYOLO and Gautomatch picked particles using cryoSPARK?

11. There is no real difference between a resolution of 3.275A and 3.19A (this is less than 0.1A). It is therefore inaccurate to claim this method achieves better resolution than the best published resolution.

Analysis of generalization ability

12. What micrographs are used to train the cross-CASSPER model?

Response to referee's comments

We thank all the three reviewers for their time and critical comments. We believe addressing them has substantially improved the quality and presentation of our manuscript.

Reviewer #1 (Remarks to the Author):

The manuscript by George et al presents a new method named CASSPER, which applies a deep learning-based semantic segmentation for automated particle picking and selection from EM micrographs. The authors tested their method using a number of experimental datasets and demonstrate certain improvement and potential advantage over existing methods. The results were presented in great detail, which appear to support the applicability of the proposed method, at least in the form of what is presented. This could be a welcoming contribution for the field of cryo-EM methods development.

Response: We thank the reviewer for finding our study as "welcoming contribution to the field of cryo-EM methods development".

However, several questions should be addressed before the manuscript can be accepted for publication.

(1) There is a lack of a baseline characterization of their method, and a baseline benchmark of the performance that can be used for comparison with existing methods. There is a consensus "bake-off" dataset (the KLH dataset available through http://emg.nysbc.org/redmine/projects/public-datasets/wiki/KLH_datasets) that has been tested for most particle picking algorithms developed so far. Providing testing results on this standard dataset presents a baseline benchmark that can be readily used for comparison with other methods in literature.

Response: We are really grateful to the reviewer for suggesting this baseline characterization using the KLH dataset. We have now used the KLH dataset to compare the performance of CASSPER, crYOLO and Topaz. In order to have proper comparisons, we carried out these evaluations on the same GPU workstation (specifications given below).

We trained CASSPER, crYOLO and Topaz using the same set of 17 micrographs and used the trained model to predict on other 15 micrographs.
We analysed the PR curve test for the three classifiers based on the test criteria of crYOLO and TOPAZ. Predicted particles were compared with manually picked particles (ground truth). The results are summarized in the table below and show that CASSPER performance is at par with crYOLO and TOPAZ.

Tool	True Positives (TP)	False Positives (FP)	False Negatives (FN)	Precision	Recall
crYOLO (Best threshold :0.638)	421	38	24	0.92	0.95
TOPAZ (Threshold score :10.69)	305	6	101	0.98	0.75
CASSPER (Optimim size of contour : 4718)	410	37	35	0.92	0.92

We have performed the test for the PR curve based on the methods described in respective tools. The optimum value of the threshold, obtained from precision recall curve, corresponds to the best F1 score. crYOLO has given the threshold value as 0.638 which corresponds to the best F1 score and we have extracted the coordinates at this optimum threshold and used it for calculating the precision and recall scores.
The Topaz test for precision recall curve shows that optimum value of threshold calculated, based on the method mentioned in the Github page of Topaz, corresponds to the associated score of 10.69. Similarly, in case of CASSPER the optimum value of size of the connected pixels is found to be 4718 and the coordinates are extracted based on this value for finding the precision and recall.

However , when we had done using full set of coordinates extracted using Topaz, the scores are as follows:
TP: 375 FP: 46 FN: 31 Precision: 0.89 Recall: 0.92

(2) The authors should present the precision-recall curves of their tests, particularly using the consensus "bake-out" KLH dataset. Otherwise, it is hard to know to what degree the implementation improves over a large number of existing methods. The comparison of final 3D maps among selected methods is useful but it is also affected by other steps in data processing, making it hard to know the net performance difference in particle picking step.

Response: As per the reviewers suggestions we carried out CASSPER Evaluation and comparison with crYOLO and Topaz using the KLH Dataset. A desktop computer with NVIDIA GeForce GTX 1070 graphics card with 64 GB memory and an Intel(R) Xeon(R) CPU was used to train and predict the particles.
We trained all the three tools using a set of 17 high defocus micrographs and predicted particles on the same set of 15 raw micrographs using the three methods. The results are tabulated below:

Precision and recall

For obtaining the precision recall curves, we made the ground truth labels by manual annotation by an expert. Trained models of all the three tools were used to predict both high defocus and low defocus micrographs. The performance of CASSPER in terms of Precision Recall and processing speed has been tabulated as follows:

Model trained using **17 high defocus micrographs**			
**Predicted for 15 micrographs**	**AUC**	**Precision**	**Recall**
High defocus	0.954	0.944	0.948
Low defocus	0.92	0.90	0.898

Processing speed tested on 15 micrographs

CASSPER	18 sec
CRYOLO	25 sec
Topaz	13.8 sec

More details are added in paper and Supplementary Information (Supplementary figure 3)

(3) How many particles are needed for training data? What is the lower and higher bound of the size of the training dataset?

Response: In CASSPER, manual picking of particles is not needed at any stage. The Semantically segmented labels are given as the training data. The minimum number of micrographs required for training depends on the possible orientations, the shape of the protein, and the relative coverage of the particles in the micrographs. The relative coverage of KLH is 19 %, and that of TcdA1 is 13 %. Thus it is observed that training with at least twelve micrographs was required for KLH, whereas training with twenty micrographs was required for TcdA1 to get a prediction AUC above 0.9. We have now added this analysis in the results section along with Figure 9.

(4) How is the approach performed compared to another deep-learning-based particle picking method Topaz (Nature Methods 16, 1153, 2019)?

Response: Topaz is a deep learning method in which training data is prepared by manually picking the positive labels i.e., the particles, while CASSPER eliminates manual particle picking.

In order to compare CASSPER with Topaz, we trained CASSPER and Topaz on the KLH dataset on the same set of 17 micrographs. We then used the trained model to predict on another set of 15 KLH micrographs. Our analysis of the PR curve test of respective methods, shows that CASSPER coordinates had precision and recall values of 0.91 and 0.92 whereas Topaz coordinates had precision and recall values of 0.89 and 0.92 respectively.

Also, the EMPIAR 10028 micrographs predicted using CASSPER cross model showed an average precision of 0.94 where Topaz had an average precision of 0.77 (Nature Methods 16, 1153, 2019). The processing speeds of Topaz and CASSPER were also comparable and are mentioned in the text and mentioned in the supplementary information section.

(5) The grey scale of raw micrographs in Figure are not properly presented. It is either too bright or too dark.

Response: We have revised the figure with the corrected grey scale of the raw micrographs.

(6) The Methods section includes insufficient details to assess the soundness of the tests and thus validity of the method. Can the authors provide the precision-recall curves for their tests on the generalized model for cross-molecule picking as well as details about the tests?

Response: We have now extensively revised our "methods" section to include the test details. The PR curves for different datasets are given in paper and the tests for PR Curve is explained in the methods section in the manuscript.

(7) How efficient is the authors' implementation? How is their computational efficiency compared to other methods?

Response: We have now carried out this comparison for CASSPER, crYOLO and Topaz on the same GPU workstation using the KLH dataset. Our results show that processing speed of CASSPER is at par with that of crYOLO and Topaz. The results are now discussed in the text and Supplementary Table 3.

(8) How does this method behave over a large range of SNRs due to variation in particle size and defocus? What is the lowest SNRs that the method can afford?

Response: We have performed an experiment on EMPIAR 10028 dataset to understand how CASSPER behaves over a large range of SNRs. Different levels of Gaussian and Poisson noise were added to the micrograph and were predicted using the cross model. The PR curve was drawn and AUC and average precision were calculated for different noise levels. (Details are given in paper, Figure 7 and in the Supplementary information section.) The AUC values stayed above 0.90 for -13 dB and -16 dB levels of Gaussian and Poisson noise respectively.

Also, we trained a model with KLH high defocus micrograph and predicted the low defocus micrograph. Results are summarized in Supplementary Table 4

The experiments show that CASSPER can perform well over a large range of SNR and defocus values.

(9) On page 5, the authors claim that "CASSPER is the first method to carry out labelling and prediction of different kinds of particles in a micrograph". This may not be true. Even the earliest computational method in particle picking has to have some ability in labelling and prediction of different kinds of particles. The only difference is the precision and accuracy. I suggest that the authors revised this sentence to "CASSPER is the first Semantic Segmentation-based method to … in a EM micrograph".

Response: We are grateful for the suggestion and have corrected it accordingly.

(10) In the introduction, the authors described Gautomatch, which was based on FindEM, as "template-free methods". This is wrong. FindEM/Gautomatch was one of the early methods based on template matching algorithm.

Response: We are grateful to the reviewer for pointing this out. We have corrected this inadvertent error.

Reviewer #2 (Remarks to the Author):

George et al present a deep-learning based program for automatic particle picking in electron micrographs. They argue the need for better programs and compare the performance of their package, named CASSPER, with two other programs used in the field (gautomatch and cryolo) in terms of number of particles found within a (subset of) 4 empiar datasets as well as the resolution of a 3d reconstruction obtained from a subset of these particles. Table 1 provides the numbers for this comparison. In bold, the authors highlight that the best resolution was obtained, for three out of the four data sets, when using CASSPER for initial particle picking. Their program uses a pixel-level classification and can produce coloured micrograph representations in which ice, protein, and carbon are classified differently (figure 2). Figure 3C shows the picking result of the three different programs for an individual micrograph from the each of the four data sets. In green, areas have been highlighted where the other two progs pick up false positives, whereas CASSPER correctly did not pick particles.

Response: We are grateful to the reviewer for his time and comments.

The introduction contains a number of claims highlighting the need for a new particle picking scheme, such as "One of the obstacles that still remains unresolved", "picking, and selection is a very tedious and challenging process", "laborious, slow, manual or semiautomated methods", "However, these tools are prone to picking huge amount of contaminants, background, and ice, and do not work optimally", "manual particle picking usually introduces a strong template bias". I must have manually picked tens to hundreds of thousands of particles in my career, and although that was sometimes laborious indeed, it was not necessarily slow within a wider scheme of solving a novel structure. Contaminations, ice and background can be taken care of already (by adjusting data collection schemes, picking parameters, templates, and 2d classification), and template bias can also be used to own's advantage if a micrograph contains different particles that one wish to separate as early as possible. It occurred not seldom to me that people who I introduced new into cryo-EM, directly wished to write a new program to improve on the particle picking process. Personally, I am not convinced that that is the biggest challenges within our field that we need to overcome right now. Their most convincing argument is, imo, "A fast, automatic method that can replace the manual processing is thus a necessity for automating the structure determination process."

Response: We have modified our introduction to emphasize the process of manual picking being tedious for "large-sized datasets" that have become a norm these days. We thank the reviewer for finding our argument regarding the need of "a fast automatic method that can replace manual picking for automating the structure determination process" convincing.

Smart microscopy would require direct feedback of the premise of the data while it is being collected. Automated particle picking would indeed be mandatory for this. Unfortunately, the manuscript does not comment on the processing speed of CASSPER. Can it keep up with automated data collection?

Response: We are grateful to the reviewer for raising this important point. We have now compared the processing speed of CASSPER with that of crYOLO and Topaz (on the same GPU workstation) for predicting four datasets(15 micrographs each). The results are now included in text and in Supplementary Table 3. Our results show that CASSPER processing speed is at par or better then that of crYOLO and Topaz. Besides, high-speed prediction clearly suggests that CASSPER can keep up with the automated data collection and be used for on-the-fly prediction.

Protein	CASSPER (seconds/mrc)	crYOLO (seconds/mrc)	Topaz (seconds/mrc)
**TcdA1**	1.92	1.87	1.87
**TRPV1**	1.76	2.23	1.5
**B-gal**	1.8	2.8	1.89
**KLH**	1.2	1.66	0.85

Within the manuscript, the prog has only been tested on empiar data sets. However, most data sets don't end up in empiar, for a wide variety of reasons. Will it also perform well for real-life data, of which only such a small percentage proved to be good enough for deposition in empiar?

Response: We thank the reviewer for his suggestion. We chose the four EMPIAR datasets as they had been used to benchmark various other cryo-EM tools in literature including for different particle picking tools as well. However, as per reviewers suggestion we also now benchmark CASSPER using the "bake-off" KLH dataset. The results are now included in section "Benchmarking CASSPER using the KLH dataset" and the results are presented in Supplementary Figure 3.
In addition we also show that CASSPER general model picks particles efficiently on GluK3 dataset (protein that we work in our lab). These are ~400kDa receptors that are asymmetric in shape. Also, the SNR is low owing to presence of detergents. We have added raw and labelled (predicted) micrographs as Supplementary figure 4

This could, for example, be evaluated by running CASSPER real-time within a cryo-EM center for a while, maybe even at ACCEM-IISc?

Response: We believe it's possible given the high processing speed of CASSPER. However, due to the ongoing COVID 19 situation we were unable to test it at a cryo-EM center. We hope

to do this in future to incorporate CASSPER in the data processing pipeline for real time evaluation of the data quality.

The authors overlooked the Nature Methods paper of Tegunov and Cramer, which was published on october 07 2019 but has been in biorxiv since jun 14 2018. It deals with real time cryo-EM processing, and does include a novel deep-learning based particle picking scheme already, named BoxNet, which is based on residual network as implemented in TensorFlow. "CASSPER uses the SS implementation by George Seif" which is also a suite in TensorFlow.

Response: We are grateful to the reviewer for pointing out this omission. We have corrected this error.

Specific comments
1. How does CASSPER perform compared to BoxNet?

Response: Unfortunately we were unable to carry out a reasonable comparison of CASSPER with Boxnet. This is because Boxnet is not available for the linux platform and currently it works only on a Windows operating system (Windows 7and above).

2. What are the differences between the two methods and why are these differences important?

Response: We summarize the difference between Boxnet and CASSPER in the table below

Sl.No.	Boxnet	CASSPER
1.	Employs ResNet architecture	Employs FRRN- an architecture based on Resnet that is highly powerful in localisation. The residual stream in FRRN is providing localization ability which other networks dont have. FRRN have residual stream in addition to pooling stream, which is the only convolutional network in Residual. By using a set of Full Resolution Residual Units (FRRU) to merge the residual stream and the information from the pooling layers at each stage, localization as well as classification accuracy during reconstruction, is ensured.
2.	Both are implemented in tensorflow	

3. Could CASSPER be used for real-time cryo-EM particle picking?

Response: As explained above, the high processing speed of CASSPER makes it amenable for real-time cryo-EM particle picking

4. A 80s ribosome is not a protein

Response: Corrected

5. The authors write "The ultimate goal of macromolecular structure determination is to explore biologically relevant intramolecular and intermolecular interactions in its native environment." Could CASSPER also be used for picking particles in tomograms of macromolecular complexes within their native, cellular environment?

Response: The current architecture of CASSPER allows the user to label aligned composite tomograms. However, we believe that certain modifications need to be introduced at the preprocessing level and also in the labelling tool so as to improve the performance of CASSPER on tomograms. This is something that we plan to incorporate in the future versions of CASSPER.

6. Surely, it should be possible to find more particles in TcdA1 using gautomatch (table 1)?
&
7. Idem for beta-gal

Response: Yes, we agree however for this comparison we used the default basic command for gautomatch (explained in methods) including the essential parameters only. More particles could be picked by iterative refinement of the Gautomatch parameters.

8. Is the resolution of a final 3d reconstruction the best criterium to evaluate CASSPER?

Response: We agree with the reviewer that the resolution of the 3D reconstruction is just an indirect measure of CASSPER performance. Thus, we carried out additional benchmarking tests using KLH "bake-off" dataset and report the precision and recall curves for the evaluation of performance.

Reviewer #3 (Remarks to the Author):

This paper introduces a new method for particle picking based on deep learning. I am unaware of another paper that suggests the use of a full-resolution residual network. This method is shown to achieve results comparable to state-of-the-art methods on two datasets, and provides a nice experiment showing generalization. I believe that this paper will be of interest to the cryo-electron microscopy community. However, I do have some questions about this method and its general applicability.

Response: We thank the reviewer for his time and for pointing out that our study is unique in "use of a full-resolution residual network" and that the achieved results are "comparable to state-of-the-art-methods"

My main concern is that the authors claim it is vital to accurately label each pixel in the training set. This is known to be difficult (if not impossible). The high levels of noise present in the micrograph cause edge-detection-based methods to fail. Furthermore, the contrast transfer function causes the projection to seem to exist beyond its edges. How are these issues dealt with?

Response: We thank the reviewer for highlighting this. Infact, the edges of the particle stacks are not taken into account for picking. This method is only limited by the intrinsic differences in protein scattering density that may cause fragmentation in the label for a single protein structure. Since this can be corrected visually, we allow the user to specify a size threshold

based on the labels predicted by CASSPER before it is used to count and pick individual particles. The centroids of the particles are then fed as star coordinates for Relion.
For reliable determination of the location of the protein particle, the pixel based protein classification does better, as it is not affected by the limitations of edge detection or shape identification methods. Even though it is possible that some pixels may be missed out or may have some projection effects, a simple eroding of the detected region can help reliably determine the centroid of the particle that is finally fed as coordinates to Relion.

Additionally, I have some questions about the results:

1. In the evaluation the authors explain that "12 micrographs that do not constitute the training dataset were randomly selected… and mean values of F1, IoU and accuracy scores… were obtained by comparing predicted labels with the corresponding ground truth". How were the ground truth values obtained?

Response: Published particle annotations were used as ground truth to evaluate the quality of the predicted labels., manually annotated labels by experts were used in case of datasets where particle annotations were unavailable. We have added this clarification in the methods section as well.

2. Some of the experiments achieve rather bad resolutions (for example, the EMPIAR 10017 dataset which is often used to test methods of particle picking). Can these results be explained?

Response: Here 10017 (Bgal) was subjected to uniform pipeline only. The uniform pipeline consists of only 1 step of 2D classification and particle selection followed by *ab initio* 3D reconstruction and homogenous refinement. That is why the resolution is lesser than the published structure**.** Indeed as pointed out by reviewer, higher resolution could be achieved by additional steps for particle cleanup and 3D refinements.

Other questions I feel should be answered in the manuscript are:

Introduction:

3. The authors claim that "the exposure differences, noise levels and variable ice thickness in micrographs also limits the performance of these tools". First, it was unclear to me which tools are meant in this sentence. Second, it is unclear to me what this claim is based on. Lastly, what effect do these factors have on the suggested particle picker?

Response: We thank the reviewer for this comment and realize that our point was not clear in the text. We meant "automatic particle pickers" in general by tools. Also, SNR of the protein particles are affected by the exposure differences (amount of dose), noise levels and variable ice thickness in micrographs. This in turn affects the performance of particle pickers. Infact, we now add an additional figure, table and description where we evaluate the performance of CASSPER on a set of micrographs after introducing different levels of Gaussian and Poisson noise.

4. The authors claim that CASSPER uses the "transmittance of the medium". What is meant by transmittance? The scattering density?

Response: We thank the reviewer for pointing this out. We have replaced "transmittance" of the medium with the scattering density.

5. The authors claim that CLAHE is used for particle identification. Indeed, this is the goal of the entire paper. What is the specific benefit of using CLAHE? (e.g. as opposed to other histogram equalization methods).

Response: CLAHE implements adaptive histogram equalization by dividing the image into grids of small areas called 'tiles'. To smoothen the boundaries, adjacent tiles are combined using bilinear interpolation. The contrast limiting feature, which is unique in CLAHE, eliminates noise amplification in an image by redistributing the excess pixel values in regions with high contrast to the neighboring pixels. We have added these lines to the text and have also added a new Supplementary Figure 5 to emphasize on the CLAHE features.

6. I think it should be made clear in the section that there is no manual picking involved as that is a rather large advantage of this method.

Response: We thank the reviewer for this suggestion. We have explicitly mentioned this now.

Implementation of CASSPER

7. The explanation about FRRNs is unclear and should be rewritten. For example, the function f(yn-1;wn) is used twice, once for the feed-forward network and once for the residual network. Is this the same function? Also, how is f related to g and h? and how is y related to j and k?

Response: We have rewritten the entire section to improve the clarity of explanation. Also, we have modified Figure 1 to improve the clarity.

"Uniform pipeline for comparison":

8. By CTF-estimated micrographs, do the authors mean CTF corrected?

Response: We meant CTF-estimated micrographs. However, CASSPER can pick particles from just motion-corrected micrographs as well.

High-resolution 3D reconstruction

9. The resolution of TRPV1 and TcdA1 published in EMDB should be added.

Response: Resolution values have been added as suggested.

10. It is unclear to me why the authors report two different resolutions for TRPV1 and TcdA1 since both reconstructions were achieved from the same picked particles. That is, since cryoSPARK produces the best results in the experiment, why report the resolution achieved by a different method in the first place? also, why weren't the molecules reconstructed from the crYOLO and Gautomatch picked particles using cryoSPARK?

Response: We thank the reviewer for pointing this out. We apologise for our description not being clear. In the first section, CASSPER manuscript talks about the uniform pipeline approach where we have obtained particles from different tools and fed in this pipeline having a fixed number of steps for all the cases. The processing steps do not involve elimination of the junk or false positive particles since there is just one round of 2D classification followed by a single selection step of good 2D class averages, ab initio reconstruction and homogenous 3D refinement. In this case we can directly correlate the resolution to the quality of particles picked since the particle set from each tool underwent the same channel.
Further, in order to demonstrate that CASSPER picked particles can achieve high-resolution, we chose two (TRPV1 and TcdA1) of the four datasets and subjected to additional steps of data cleanup and 3D refinements including non-uniform refinement and local refinement as implemented in cryoSPARC. This led to higher resolution reconstruction.

CASSPER, crYOLO and Gautomatch were subjected to uniform pipeline and only two datasets from CASSPER picked particles were taken to higher-resolution reconstruction following further refinements.

11. There is no real difference between a resolution of 3.275A and 3.19A (this is less than 0.1A). It is therefore inaccurate to claim this method achieves better resolution than the best published resolution.

Response: Indeed there is no difference when we look at the values but if we compare our Trpv1 map with the published one we can clearly point out that we have obtained a better quality map which entails finer features.

Analysis of generalization ability

12. What micrographs are used to train the cross-CASSPER model?

Response: We have now included a Supplementary table showing the list of training dataset.

180 micrographs from 13 different protein sets are used for training the cross model.

1. EMPIAR-10272 (horse spleen apoferritin)
2. EMPIAR-10025(T20S Proteasome)
3. EMPIAR-10096(Influenza Hemagglutinin Trimer)
4. EMPIAR-10175(hemagglutinin)
5. EMPIAR-10215(Rabbit muscle aldolase)
6. EMPIAR-10217(bovine liver glutamate dehydrogenase)
7. EMPIAR-10285(P-Rex1–G-beta-gamma signaling scaffold)
8. EMPIAR-10168(RNA Polymerase III pre-initiation complex)
9. EMPIAR-10208(mouse MDA5-dsRNA 10mM ATP Filament)
10. EMPIAR-10081(human HCN1 hyperpolarization-activated cyclic nucleotide-gated ion channel)
11. EMPIAR-10005(TRPV1)
12. KLH Dataset
13. EMPIAR-10099(Hrd1 and Hrd3 complex)

REVIEWERS' COMMENTS:

Reviewer #1 (Remarks to the Author):

The authors have addressed all my questions. I'd like to recommend its publication.

Reviewer #3 (Remarks to the Author):

This paper presents a novel method for particle picking. The method is based on a full-resolution residual network and is comparable to state-of-the-art particle pickers. I feel this work will be of interest to the Cryo-EM community.

In my last review I have raised a number of questions concerning the manuscript. These have now been addressed.

I note that the comparison with other methods is based on a uniform pipeline. While, in my opinion, this is sufficient and demonstrates the potential of the suggested method, using a minimal number of steps may not be a completely fair comparison since, for structure-driven drug design, we are interested in resolving structures at very high resolutions.

We sincerely thank the reviewers for supporting our study. They have been very constructive and extremely useful for improving our manuscript. We also thank you and the editorial staff for providing detailed editorial comments and check list.

REVIEWERS' COMMENTS:

Reviewer #1 (Remarks to the Author):

The authors have addressed all my questions. I'd like to recommend its publication.

Response: We thank the reviewer for supporting our study and recommending its publication.

Reviewer #3 (Remarks to the Author):

This paper presents a novel method for particle picking. The method is based on a full-resolution residual network and is comparable to state-of-the-art particle pickers. I feel this work will be of interest to the Cryo-EM community.

Response: We thank the reviewer for supporting our study.

In my last review I have raised a number of questions concerning the manuscript. These have now been addressed.

Response: We thank the reviewer for his time and comments. They have been very useful in improving our manuscript.

I note that the comparison with other methods is based on a uniform pipeline. While, in my opinion, this is sufficient and demonstrates the potential of the suggested method, using a minimal number of steps may not be a completely fair comparison since, for structure-driven drug design, we are interested in resolving structures at very high resolutions.

Response: CASSPER is fully capable of picking particle that lead to high-resolution structure determination. The uniform pipeline is only used for initial stages to save on computation time and resources. We demonstrate that CASSPER picked particle can give high-resolution structures as presented in Figure 4 and Figure 5 for TcdA1 and TRPV1 datasets. These are also discussed in main text on pages 12 and 13 under section “High-resolution 3D reconstruction”. Further, our other measurements demonstrate that CASSPER performs either at par or better than existing tools like crYOLO and Topaz.
